# Triterpenoids from the Leaves of *Diospyros digyna* and Their PTP1B Inhibitory Activity

**DOI:** 10.3390/molecules29071640

**Published:** 2024-04-05

**Authors:** Lan Huang, Ziqi Wang, Fangxin Wang, Song Wang, Dezhi Wang, Meihua Gao, Hua Li, Min Song, Xiaoqi Zhang

**Affiliations:** 1 Guangdong Provincial Engineering Research Center for Modernization of TCM, Jinan University, Guangzhou 510632, China; huanglan@stu2021.jnu.edu.cn (L.H.); wangziqi@stu2021.jnu.edu.cn (Z.W.); wfxwfx@stu2022.jnu.edu.cn (F.W.); wangsong@stu2022.jnu.edu.cn (S.W.); dezhiwoo125@stu2020.jnu.edu.cn (D.W.); songm1017@jnu.edu.cn (M.S.); 2 NMPA Key Laboratory for Quality Evaluation of TCM, Jinan University, Guangzhou 510632, China; 3 Guangdong Institute for Drug Control, Guangzhou 510663, China; gaomeihua@gdidc.org.cn (M.G.); bgs@gdidc.org.cn (H.L.)

**Keywords:** *Diospyros digyna*, Ebenaceae, ursane triterpenoids, PTP1B inhibitory activity

## Abstract

Six new 2*α*-hydroxy ursane triterpenoids, 3*α*-*cis*-*p*-coumaroyloxy-2*α*,19*α*-dihydroxy-12-ursen-28-oic acid (**1**), 3*α-trans*-*p*-coumaroyloxy-2*α*,19*α*-dihydroxy-12-ursen-28-oic acid (**2**), 3*α*-*trans*-*p*-coumaroyloxy-2*α*-hydroxy-12-ursen-28-oic acid (**3**), 3*β-trans*-*p*-coumaroyloxy-2*α*-hydroxy-12,20(30)-ursadien-28-oic acid (**4**), 3*β-trans*-feruloyloxy-2*α*-hydroxy-12,20(30)-ursadien-28-oic acid (**5**), and 3*α-trans*-feruloyloxy-2*α*-hydroxy-12,20(30)-ursadien-28-oic acid (**6**), along with eleven known triterpenoids (**7**–**17**), were isolated from the leaves of *Diospyros digyna*. Their chemical structures were elucidated by comprehensive analysis of UV, IR, HRESIMS, and NMR spectra. All the isolated compounds were evaluated for their PTP1B inhibitory activity. 3*β*-*O*-*trans*-feruloyl-2*α*-hydroxy-urs-12-en-28-oic acid (**13**) showed the best inhibition activity with an IC_50_ value of 10.32 ± 1.21 μM. The molecular docking study found that the binding affinity of compound **13** for PTP1B was comparable to that of oleanolic acid (positive control).

## 1. Introduction

Type 2 diabetes (T2DM), a chronic metabolic disease, primarily results from an impaired insulin receptor signaling pathway [1]. T2DM accounts for approximately 90% of cases of diabetes. The multifactorial etiology of T2DM, including genetic, environmental, and lifestyle factors, has made its management and prevention a significant challenge for healthcare systems worldwide [2]. Protein tyrosine phosphatase 1B (PTP1B), an intracellular enzyme, has been implicated in the negative regulation of insulin signaling [3]. The overexpression of PTP1B in various tissues of T2DM patients highlights its pivotal role in the pathogenesis of insulin resistance [4]. Recent studies have demonstrated that genetic deletion or the pharmacological inhibition of PTP1B enhances insulin sensitivity and protects against diet-induced obesity [5,6,7,8], thereby underscoring PTP1B as a crucial therapeutical target for T2DM. Several PTP1B inhibitors have shown promise in preclinical models by improving glycemic control and insulin sensitivity [9]. Natural products are a rich source of new drug candidates [10] and an important source of PTP1B inhibitors [11,12].

**Figure 1:**
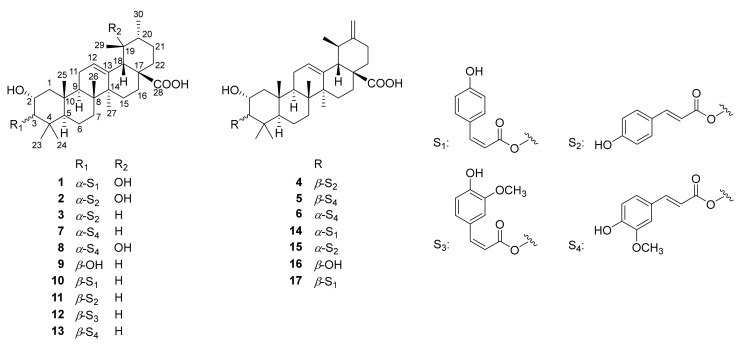
Chemical structures of **1**–**17**.

The *Diospyros* genus comprises over 500 species of evergreen trees and shrubs, belonging to the family of Ebenaceae [13]. *Diospyros* plants are widely distributed in pantropical regions, and some species (particularly *Diospyros digyna* Jacq.) are edible fruit-yielding plants [14]. The chemical constituents of the plants, including triterpenoids, flavonoids, tannins, sugars, and phenolic acids, show antioxidant, anti-inflammatory, antiviral, antitumor, and PTP1B inhibitory activities [15,16,17]. In the search for natural PTP1B inhibitors, a phytochemical investigation on the leaves of *D. digyna* was carried out. Six new 2*α*-hydroxy ursane triterpenoids (**1**–**6**), along with eleven known ones were isolated from this plant (Figure 1). In addition, the PTP1B inhibitory activity of these triterpenoids was tested. Herein, the isolation, structural elucidation, and bioactivity of the isolates are presented.

## 2. Results

Compound **1** was isolated as a white amorphous powder. Its molecular formula was deduced as C_39_H_54_O_7_ based on its HRESIMS ion at *m*/*z* 635.3934 [M + H]^+^. The UV spectrum showed the absorption maxima at 206, 228, and 310 nm. The IR spectrum suggested the presence of hydroxy (3425 cm^−1^), carbonyl (1694 cm^−1^), and aromatic (1605, 1513, and 1454 cm^−1^) groups. The ^1^H and ^13^C NMR spectra showed a *cis*-*p*-coumaroyl group [*δ*_H_ 7.66 (2H, d, *J* = 8.7 Hz), 6.88 (1H, d, *J* = 13.0 Hz), 6.75 (2H, d, *J* = 8.7 Hz), 5.87 (1H, d, *J* = 13.0 Hz); *δ*_C_ 168.6, 160.0, 144.8, 133.8 (×2), 127.9, 117.5, 116.0 (×2)], an olefinic bond [*δ*_H_ 5.32 (1H, m); *δ*_C_ 140.2, 129.4], two oxymethines [*δ*_H_ 5.00 (1H, d, *J* = 4.1 Hz), 4.11 (1H, dt, *J* = 11.0, 4.1 Hz); *δ*_C_ 81.4, 66.2], and seven methyls [*δ*_H_ 1.36 (3H, s), 1.23 (3H, s), 1.04 (3H, s), 1.00 (3H, s), 0.95 (3H, d, *J* = 6.7 Hz), 0.91 (3H, s), 0.81 (3H, s); *δ*_C_ 28.7, 27.2, 25.1, 22.4, 17.7, 17.0, 16.8] (Table 1). Comparison of the 1D NMR spectra of **1** with those of 3-*O*-*cis*-*p*-coumaroyltormentic acid [18] indicated similar planar structures, which was further verified by the 2D NMR spectra of **1**.

**Figure 2:**
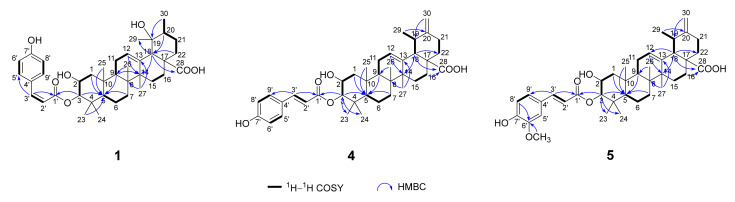
Key ^1^H–^1^H COSY and HMBC correlations of **1**, **4**, and **5**.

**Figure 3:**
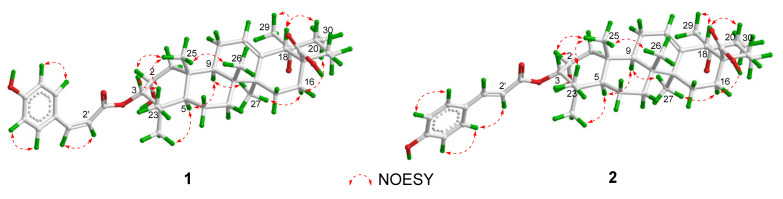
Key NOESY correlations of **1** and **2**.

The ^1^H–^1^H COSY spectrum of **1** suggested spin-coupling systems of H_2_-1/H-2/H-3, H-5/H_2_-6/H_2_-7, H-9/H_2_-11/H-12, H_2_-15/H_2_-16, H_3_-30/H-20/H_2_-21/H_2_-22, H-2′/H-3′, H-5′/H-6′, and H-8′/H-9′ (Figure 2). In the HMBC spectrum, the correlations from H_2_-1/H-3/H_2_-7/H-9/H_3_-25 to C-5, from H-9/H-12/H-18 to C-14, from H-18 to C-12/C-14/C-16/C-28/C-29, from H-20/H-22 to C-18, from H_3_-23 to C-3/C-5/C-24, from H_3_-25 to C-1/C-5/C-9, from H_3_-26 to C-7/C-9, from H_3_-27 to C-8/C-13/C-15, and from H_3_-30 to C-19/C-21, established an ursane-type triterpenoid skeleton (Figure 2). Moreover, the HMBC correlation from H-3 to C-1′ located the *cis*-*p*-coumaroyl group at C-3. In the NOESY spectrum, the correlations between H-9/H_3_-24 and H-5, between H-9/H-16*α* and H_3_-27, and between H-16*α* and H_3_-30 suggested H-5, H-9, H_3_-24, and H_3_-27 were *α*-oriented. The NOE correlations between H-2/H_3_-23/H_3_-26 and H_3_-25, between H-3 and H_3_-23, and between H-20/H_3_-29 and H-18 suggested H-2, H-3, H-18, H-20, H_3_-23, H_3_-25, H_3_-26, and H_3_-29 were *β*-oriented (Figure 3). Thus, the structure of **1** was elucidated and named as 3*α*-*cis*-*p*-coumaroyloxy-2*α*,19*α*-dihydroxy-12-ursen-28-oic acid.

The molecular formula of **2** was determined as C_39_H_54_O_7_ by the HRESIMS at *m*/*z* 635.3938 [M + H]^+^ (calcd for C_39_H_55_O_7_, 635.3942). The UV spectrum showed the absorption maxima at 205, 226, and 312 nm. The IR spectrum suggested the presence of hydroxy (3417 cm^−1^), carbonyl (1693 cm^−1^), and aromatic (1608, 1515, and 1453 cm^−1^) groups. The 1D NMR data of **2** were similar to those of **1**, except for the presence of a *trans*-*p*-coumaroyl group [*δ*_H_ 7.47 (2H, d, *J* = 8.6 Hz), 7.63 (1H, d, *J* = 15.9 Hz), 6.82 (2H, d, *J* = 8.6 Hz), 6.40 (1H, d, *J* = 15.9 Hz); *δ*_C_ 169.5, 161.3, 146.4, 131.3 (×2), 127.4, 117.0 (×2), 116.0], and the absence of a *cis*-*p*-coumaroyl group (Table 1). The structure of **2** was verified by its 2D NMR spectra. In the NOESY spectrum, the correlations between H-3/H_3_-25 and H_3_-23 indicated that H-3 was *β*-oriented (Figure 3). Thus, the structure of **2** was elucidated and named as 3*α-O*-*trans*-*p*-coumaroyloxy-2*α*,19*α*-dihydroxy-12-ursen-28-oic acid.

The molecular formula of **3** was determined as C_39_H_54_O_6_ by the HRESIMS at *m*/*z* 619.3986 [M + H]^+^ (calcd for C_39_H_55_O_6_, 619.3993). The UV spectrum showed the absorption maxima at 208, 228, and 312 nm. The IR spectrum suggested the presence of hydroxy (3449 cm^−1^), carbonyl (1695 cm^−1^), and aromatic (1599, 1519, and 1458 cm^−1^) groups. The 1D NMR data of **3** were similar to those of jacoumaric acid (**11**) [19,20], except for the chemical shifts of Δ*δ*_C_ −5.0 (C-1), −1.7 (C-2), −4.1 (C-3), −1.6 (C-4), −5.1 (C-5), −0.6 (C-23), and +3.7 (C-24), which indicated **3** might be a 3-epimer of **11**. In the NOESY spectrum, the similar correlations between H-3/H_3_-25 and H_3_-23 suggested that H-3 was *β*-oriented. Based on the above analysis, compound **3** was determined to be 3*α*-*O*-*trans*-*p*-coumaroyloxy-2*α*-hydroxy-12-ursen-28-oic acid.

**Figure 4:**
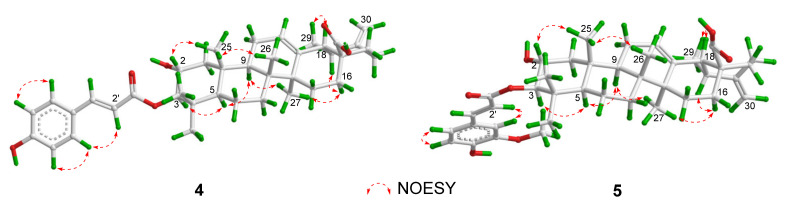
Key NOESY correlations of **4** and **5**.

Compound **4** was isolated as an amorphous powder. Its HRESIMS at *m*/*z* 639.3637 [M + Na]^+^ exhibited the molecular formula of C_39_H_52_O_6_. The UV spectrum showed the absorption maxima at 208, 228, and 312 nm. The IR spectrum suggested the presence of hydroxy (3204 cm^−1^), carbonyl (1695 cm^−1^), and aromatic (1602, 1515, and 1453 cm^−1^) groups. The ^1^H and ^13^C NMR spectra showed a *trans*-*p*-coumaroyl group [*δ*_H_ 8.02 (1H, d, *J* = 15.9 Hz), 7.57 (2H, d, *J* = 8.5 Hz), 7.18 (2H, d, *J* = 8.5 Hz), 6.70 (1H, d, *J* = 15.9 Hz); *δ*_C_ 168.4, 161.8, 145.3, 131.1, 131.1, 126.7, 117.3, 117.3, 116.6], two olefinic bonds [*δ*_H_ 5.47 (1H, m), 4.84 (1H, br s), 4.79 (1H, br s); *δ*_C_ 154.2, 139.5, 126.2, 105.6], two oxymethines [*δ*_H_ 5.28 (1H, d, *J* = 10.8 Hz), 4.32 (1H, ddd, *J* = 10.8, 4.3, 3.7 Hz); *δ*_C_ 85.5, 66.8], and six methyls [*δ*_H_ 1.22 (3H, s), 1.13 (3H, d, *J* = 6.4 Hz), 1.09 (3H, s), 1.06 (3H, s), 1.03 (3H, s), 1.00 (3H, s); *δ*_C_ 29.5, 24.2, 18.7, 17.9, 17.4, 17.1] (Table 2). The above NMR data of **4** were similar to those of 3*α-trans*-coumaroyloxy-2*α*-hydroxy-12,20(30)-dien-28-ursolic acid (**15**) [21], except for the chemical shifts of Δ*δ*_C_ +5.0 (C-1), +1.7 (C-2), +4.1 (C-3), +1.3 (C-4), +5.1 (C-5), +0.7 (C-23), and −3.7 (C-24), which indicated **4** might be a 3-epimer of **15**. In the NOESY spectrum, the correlations between H-2 and H_3_-25, and between H-3 and H-5, suggested that H-2 was *β*-oriented, while H-3 was *α*-oriented (Figure 4). Thus, the structure of **4** was elucidated and named as 3*β-trans*-*p*-coumaroyloxy-2*α*-hydroxy-12,20(30)-ursadien-28-oic acid.

The molecular formula of **5** was determined as C_40_H_54_O_7_ by the HRESIMS at *m*/*z* 647.3944 [M + H]^+^ (calcd for C_40_H_55_O_7_, 647.3942). The UV spectrum showed the absorption maxima at 208, 238, and 325 nm. The IR spectrum suggested the presence of hydroxy (3301 cm^−1^), carbonyl (1699 cm^−1^), and aromatic (1598, 1514, and 1447 cm^−1^) groups. Comparison of NMR data of **5** to those of **4** (Table 2) revealed similar structures, except for the presence of a methoxy group [*δ*_H_ 3.80 (3H, s); *δ*_C_ 56.4] in **5**. In the HMBC spectrum, the correlation from CH_3_O- to C-6′ located CH_3_O- at C-6′ (Figure 2). Thus, the structure of **5** was elucidated and named as 3*β-trans*-feruloyloxy-2*α*-hydroxy-12,20(30)-ursadien-28-oic acid.

The molecule formula of **6** was identical to that of **5** based on the HRESIMS at *m/z* 647.3945 [M + H]^+^ (calcd for C_40_H_55_O_7_, 647.3942). The UV spectrum showed the absorption maxima at 208, 242, and 325 nm. The IR spectrum suggested the presence of hydroxy (3450 cm^−1^), carbonyl (1699 cm^−1^), and aromatic (1597, 1517, and 1460 cm^−1^) groups. The 1D NMR data of **6** were similar to those of **5**, except for the chemical shifts of Δ*δ*_C_ +5.0 (C-1), +1.8 (C-2), +4.1 (C-3), +1.1 (C-4), +5.2 (C-5), +0.6 (C-23), and −3.7 (C-24), which indicated **6** might be a 3-epimer of **5**. In the NOESY spectrum, the correlations between H-3/H_3_-25 and H_3_-23 suggested that H-3 was *β*-oriented. Thus, the structure of **6** was identified and named as 3*α-trans*-feruloyloxy-2*α*-hydroxy-12,20(30)-ursadien-28-oic acid.

Apart from the above six new 2*α*-hydroxy ursane triterpenoids (**1**–**6**), eleven known triterpenoids were isolated and identified as 3*α*-trans-feruloyloxy-2*α*-hydroxyurs-12-en-28-oic acid (**7**) [22], 3-*O*-*trans*-feruloyl euscaphic acid (**8**) [23], colosolic acid (**9**) [24], 3*β*-*O*-*cis*-*p*-coumaroyl-2*α*-hydroxy-urs-12-en-28-oic acid (**10**) [20,25], jacoumaric acid (**11**) [20], 3*β*-*O*-*cis*-feruloyl-2*α*-hydroxy-urs-12-en-28-oic acid (**12**) [20], 3*β*-*O*-*trans*-feruloyl-2*α*-hydroxy-urs-12-en-28-oic acid (**13**) [20], (2*α*,3*α*)-2-hydroxy-3-[(2*Z*)-3-(4-hydroxyphenyl)-1-oxo-2-propenyl]oxy]ursa-12,20(30)-dien-28-oic acid (**14**) [21], 3*α-trans*-coumaroyloxy-2*α*-hydroxy-12,20(30)-dien-28-ursolic acid (**15**) [21], 2α-hydroxymicromeric acid (**16**) [26], and 3*β*-*cis*-*p*-coumaroyloxy-2*α*-hydroxyursa-12,20(30)-dien-28-oic acid (**17**) [27].

**Figure 5:**
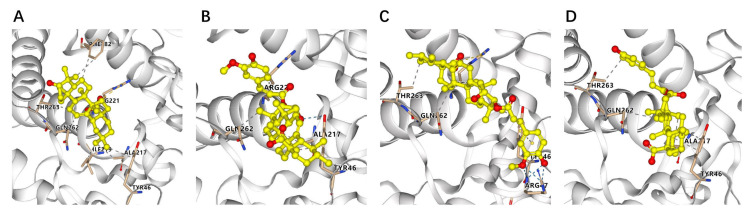
Three-dimensional ligand interaction diagrams of oleanolic acid (**A**), **12** (**B**), **13** (**C**), and **15** (**D**) at the active site of PTP1B enzyme (blue dashed lines indicate hydrogen bond, gray dashed lines indicate hydrophobic interaction).

All the isolated compounds were evaluated for their PTP1B inhibitory activity. As a result, compounds **4**–**6**, **10**–**13**, and **15** showed PTP1B inhibition with IC_50_ values in the range of 10.32–48.67 μM (Table 3), while other compounds were over 50 μM. Compounds **12**, **13**, and **15** showed better inhibition activity with IC_50_ values of 16.20, 10.32, and 17.12 μM, respectively. To know more about the binding and interaction mode between PTP1B and compounds **12**, **13**, **15**, and oleanolic acid, a molecular docking study was conducted by AutoDock Vina. The binding energies of compounds **12**, **13**, and **15** to PTP1B are −7.1, −7.8, and −7.5 kcal/mol, respectively. Compound **13** is slightly better than that of oleanolic acid (−7.6 kcal/mol), suggesting comparable binding affinity to PTP1B. As shown in Figure 5, these active compounds can dock into the same hydrophobic pocket and bind to the catalytic residues (Gln262, Ala217, and Tyr46) by different interactions as the positive control.

## 3. Experimental Section

### 3.1. General Experimental Procedures

UV, IR, ECD, and optical rotations were recorded on JASCO V550 UV/VIS, JASCO FTR-4600, JASCO-180, and JASCO P-2000 spectrometers (JASCO, Tokyo, Japan), respectively. HRESIMS were obtained using an Agilent 6210 LC/MSD TOF mass spectrometer (Agilent Technologies, Inc., Santa Clara, CA, USA). NMR data were measured by a Bruker AV-400 NMR spectrometer (Bruker, Fällanden, Switzerland). The preparative HPLC was carried on an Agilent 1200 HPLC (Agilent Technologies, Inc., Santa Clara, CA, USA) with a Cosmosil 5C18-MS-II (250 mm × 10 mm, 5 μm). The GF_254_ silica gel plates were purchased from the Yantai Institute of Industrial Chemistry, Yantai, China. Silica gel (80–100 mesh, 100–200 mesh, and 200–300 mesh; Qingdao Marine Chemical, Ltd., Qingdao, China), ODS (C_18_, Merck, Darmstadt, Germany), and Sephadex LH-20 (Pharmacia, Kalamazoo, MI, USA) were used for column chromatography.

### 3.2. Plant Material

The leaves of *Diospyros digyna* Jacq. were collected from Zhongshan Haizaoye Agricultural Technology Co., Ltd., Guangdong, China, in July 2018. The plant was identified by Prof. Guangxiong Zhou, College of Pharmacy, Jinan University. A voucher specimen (No. CP2018070903) was deposited in the herbarium of Jinan University.

### 3.3. Extraction and Isolation

The dried leaves of *D. digyna* (17.0 kg) were extracted with 95% ethanol at room temperature to give the crude extract (4.3 kg). Then, the crude extract was suspended into H_2_O, and partitioned successively with petroleum ether (PE), ethyl acetate (EA), and *n*-butanol, respectively. The ethyl acetate extract (803 g) was subjected to a silica gel column and eluted with CH_2_Cl_2_/CH_3_OH (100:0→0:100, *v*/*v*) to obtain eight main fractions (Fr. A–Fr. H). Fr C (20 g) was subjected to a silica gel column eluted with PE/EA (10:1→1:1, *v*/*v*) to afford six subfractions (Fr. C1–Fr. C6). Fr. C3 (5.0 g) was purified by Sephadex LH-20 columns (CHCl_3_:CH_3_OH = 1:1, *v*/*v*) and preparative HPLC to afford **3** (8.5 mg, CH_3_OH:H_2_O:HCOOH = 83:17:0.1, *v*/*v*/*v*, *t*_R_ = 32.7 min), **9** (8.0 mg, CH_3_OH:H_2_O:HCOOH = 98:2:0.1, *v*/*v*/*v*, *t*_R_ = 32.5 min), **11** (9.0 mg, CH_3_OH:H_2_O:HCOOH = 85:15:0.1, *v*/*v*/*v*, *t*_R_ = 11.0 min), **13** (6.0 mg, CH_3_OH:H_2_O:HCOOH = 83:17:0.1, *v*/*v*/*v*, *t*_R_ = 31.5 min), and **14** (13.0 mg, CH_3_OH:H_2_O:HCOOH = 83:17:0.1, *v*/*v*/*v*, *t*_R_ = 39.3 min). Fr. D (15 g) was subjected to an ODS column eluted with CH_3_OH/H_2_O (100:0→0:100, *v*/*v*) to obtain seven subfractions (Fr. D1–Fr. D7). Fr. D4 (5.8 g) was purified by Sephadex LH-20 columns (CH_3_OH) and preparative HPLC to afford **1** (26.0 mg, CH_3_CN:H_2_O:HCOOH = 60:40:0.1, *v*/*v*/*v*, *t*_R_ = 22.1 min), **2** (10.0 mg, CH_3_CN:H_2_O:HCOOH = 60:40:0.1, *v*/*v*/*v*, *t*_R_ = 25.2 min), **10** (10.0 mg, CH_3_OH:H_2_O:HCOOH = 80:20:0.1, *v*/*v*/*v*, *t*_R_ = 15.5 min), **12** (20.0 mg, CH_3_CN:H_2_O:HCOOH = 75:25:0.1, *v*/*v*/*v*, *t*_R_ = 17.5 min), **16** (2.0 mg, CH_3_CN:H_2_O:HCOOH = 65:35:0.1, *v*/*v*/*v*, *t*_R_ = 20.5 min), and **17** (6.5 mg, CH_3_CN:H_2_O:HCOOH = 65:35:0.1, *v*/*v*/*v*, *t*_R_ = 22.5 min). Fr. F (10 g) was subjected to a silica gel column eluted with PE/EA (9:1→0:10, *v*/*v*) to obtain six subfractions (Fr. F1–Fr. F6). Fr. F4 (2 g) was purified by a MCI column and preparative HPLC to afford **4** (10.0 mg, CH_3_CN:H_2_O:HCOOH = 60:40:0.1, *v*/*v*/*v*, *t*_R_ = 19.5 min), **5** (12.0 mg, CH_3_CN:H_2_O:HCOOH = 60:40:0.1, *v*/*v*/*v*, *t*_R_ = 14.5 min), **6** (9.0 mg, CH_3_CN:H_2_O:HCOOH = 70:30:0.1, *v*/*v*/*v*, *t*_R_ = 32.0 min), **7** (28.5 mg, CH_3_CN:H_2_O:HCOOH = 70:30:0.1, *v*/*v*/*v*, *t*_R_ = 24.3 min), **8** (19.0 mg, CH_3_CN:H_2_O:HCOOH = 60:40:0.1, *v*/*v*/*v*, *t*_R_ = 14.0 min), and **15** (12.0 mg, CH_3_OH:H_2_O:HCOOH = 80:20:0.1, *v*/*v*/*v*, *t*_R_ = 12.5 min).

Compound **1**: white amorphous powder; ${\left[\alpha \right]}_{D}^{25}$ + 16 (*c* 1.3, CH_3_OH); UV (CH_3_OH) *λ_max_* (log *ε*) 206 (3.59), 228 (3.43), 310 (3.58) nm; IR (KBr) *v_max_* 3425, 2937, 1694, 1605, 1513, 1454, 1387, 1273, 1173, 1041, 979, 937, 839 cm^−1^; HRESIMS *m/z*: 635.3934 [M + H]^+^ (calcd for C_39_H_55_O_7_, 635.3942); ^1^H NMR (400 MHz, CD_3_OD) and ^13^C NMR (100 MHz, CD_3_OD) (Table 1 and Figures S1–S6, Supplementary Materials).

Compound **2**: white amorphous powder; ${\left[\alpha \right]}_{D}^{25}$ + 24 (*c* 1.3, CH_3_OH); UV (CH_3_OH) *λ_max_* (log *ε*) 205 (3.62), 226 (3.46), 312 (3.64) nm; IR (KBr) *v_max_* 3417, 2938, 1693, 1608, 1515, 1453, 1386, 1272, 1198, 1177, 1041, 960, 936, 837 cm^−1^; HRESIMS *m*/*z*: 635.3938 [M + H]^+^ (calcd for C_39_H_55_O_7_, 635.3942); ^1^H NMR (400 MHz, CD_3_OD) and ^13^C NMR (100 MHz, CD_3_OD) (Table 1 and Figures S10–S15, Supplementary Materials).

Compound **3**: white amorphous powder; ${\left[\alpha \right]}_{D}^{25}$ + 35 (*c* 0.3, CH_3_OH); UV (CH_3_OH) *λ_max_* (log *ε*) 208 (3.46), 228 (3.31), 312 (3.53) nm; IR (KBr) *v_max_* 3449, 2935, 1695, 1599, 1519, 1458, 1268, 1178, 1039, 984, 819 cm^−1^; HRESIMS *m*/*z*: 619.3986 [M + H]^+^ (calcd for C_39_H_55_O_6_, 619.3993); ^1^H NMR (400 MHz, C_5_D_5_N) and ^13^C NMR (100 MHz, C_5_D_5_N) (Table 1 and Figures S19–S24, Supplementary Materials).

Compound **4**: white amorphous powder; ${\left[\alpha \right]}_{D}^{25}$ + 24 (*c* 1.4, CH_3_OH); UV (CH_3_OH) *λ_max_* (log *ε*) 208 (3.49), 228 (3.30), 312 (3.54) nm; IR (KBr) *v_max_* 3204, 2938, 1695, 1602, 1515, 1453, 1385, 1273, 1175, 1042, 961, 938, 840 cm^−1^; HRESIMS *m*/*z*: 639.3637 [M + Na]^+^ (calcd for C_39_H_52_O_6_Na, 639.3656); ^1^H NMR (400 MHz, C_5_D_5_N) and ^13^C NMR (100 MHz, C_5_D_5_N) (Table 2 and Figures S28–S33, Supplementary Materials).

Compound **5**: white amorphous powder; ${\left[\alpha \right]}_{D}^{25}$ + 94 (*c* 1.7, CH_3_OH); UV (CH_3_OH) *λ_max_* (log *ε*) 208 (3.60), 238 (3.37), 325 (3.51) nm; IR (KBr) *v_max_* 3301, 2939, 1699, 1598, 1514, 1447, 1369, 1268, 1170, 1102, 1018, 869, 833 cm^−1^; HRESIMS *m*/*z*: 647.3944 [M + H]^+^ (calcd for C_40_H_55_O_7_, 647.3942); ^1^H NMR (400 MHz, C_5_D_5_N) and ^13^C NMR (100 MHz, C_5_D_5_N) (Table 2 and Figures S37–S42, Supplementary Materials).

Compound **6**: white amorphous powder; ${\left[\alpha \right]}_{D}^{25}$ + 24 (*c* 1.4, CH_3_OH); UV (CH_3_OH) *λ_max_* (log *ε*) 208 (3.48), 242 (3.26), 325 (3.35) nm; IR (KBr) *v_max_* 3450, 2942, 1699, 1597, 1517, 1460, 1378, 1269, 1175, 1131, 1037, 966, 942, 817 cm^−1^; HRESIMS *m*/*z*: 647.3945 [M + H]^+^ (calcd for C_40_H_55_O_7_, 647.3942); ^1^H NMR (400 MHz, C_5_D_5_N) and ^13^C NMR (100 MHz, C_5_D_5_N) (Table 2 and Figures S46–S51, Supplementary Materials).

### 3.4. PTP1B Inhibition Assay

The inhibitory activity of the isolated compounds against PTP1B (Abcam, Cambridge, UK, human recombinant) was assayed according to the method reported previously [28]. The reagent *p*-nitrophenyl phosphate (*p*NPP) was used as the substrate, and oleanolic acid was used as the positive control. In brief, a 100 μL assay mixture containing 1 μg/mL PTP1B, samples, 4 mM *p*NPP, 55 mM NaCl, 2.2 mM DTT, 1.1 mM EDTA, and 1 mM BSA in 11 mM Tris-HCl, pH 7.5, was incubated at 37 °C for 30 min in a 96-well plate. The absorbance of 405 nm was measured by a microplate reader. Data were analyzed by GraphPad Prism v.10.2.0 software. All data were obtained in triplicate and presented as means ± SD.

### 3.5. Molecular Docking Analysis

The crystal structure of PTP1B (PDB ID: 8U1E) was obtained from the RCSB Protein Data Bank database. The receptor was prepared by PyMOL 2.5.0 and deposited as a .pdb format. The 3D structures of the ligands were energy optimized by ChemDraw 3D 18.0 and deposited as a mol2 format. The ligands and receptors for molecular docking analysis were conducted by AutoDock Vina 1.2.2 [29]. The grid box parameters (X-center = 0.405, Y-center = 12.132, Z-center = 25.000; x-dimension = 46, y-dimension = 60, z-dimension = 48) were set to cover the binding pocket in the receptor. The docking calculation results were analyzed by PyMOL 2.5.0.

## 4. Conclusions

In summary, six new 2*α*-hydroxy ursane triterpenoids (**1**–**6**), along with eleven known ursane triterpenoids (**7**–**17**), were isolated from the leaves of *D. digyna*. Compounds **4**–**6**, **10**–**13**, and **15** showed PTP1B inhibitory activity. Notably, compound **13** demonstrated PTP1B inhibition comparable to that of oleanolic acid (positive control). The structure–activity relationships of these triterpenoids were briefly summarized. Compound **13** showed stronger PTP1B inhibitory activity than those of **10**–**12**, indicating that the *trans*-feruloyl group at C-3 strengthens the activity. For compounds **3**, **7**, **11**, and **13**, the *α*-orientation of the substituents at C-3 weakened the activity. Moreover, the molecular docking study further confirmed the binding affinity between compound **13** and PTP1B. The naturally occurring PTP1B inhibitors might reveal the potential utilization of *D. digyn* in the treatment of T2DM, and their action mechanisms deserve further investigation.

## Figures and Tables

**Table 1:** ^1^H (400 MHz) and ^13^C NMR (100 MHz) data of **1**–**3** (*δ* in ppm, *J* in Hz) *^a^.*

No.		1 *^b^*		2 *^b^*		3 *^c^*	
		*δ*_H_	*δ*_C_	*δ*_H_	*δ*_C_	*δ*_H_	*δ*_C_
1	*β*	1.65	43.2	1.71	43.5	2.05	44.1
	*α*	1.37		1.38		1.86, m	
2		4.11, dt (11.0, 4.1)	66.2	4.12, dt (10.4, 4.1)	66.2	4.50, dt (11.4, 4.3)	65.2
3		5.00, d (4.1)	81.4	5.03, d (4.1)	81.7	5.62, d (4.3)	81.4
4			39.6		39.8		39.2
5		1.19, m	51.1	1.30	51.3	1.56, m	50.9
6	*α*	1.47	19.3	1.50	19.3	1.49, m	18.8
	*β*	1.47		1.45		1.34	
7	*α*	1.59, m	34.1	1.60	34.2	1.67, m	33.8
	*β*	1.34		1.35		1.41	
8			41.4		41.4		40.6
9		1.82	48.6	1.94, m	48.8	2.00	48.6
10			39.6		39.7		39.2
11	*α*	2.04, m	24.8	2.06, m	24.9	2.12	24.2
	*β*	1.45		1.36		2.02	
12		5.32, m	129.4	5.34, m	129.4	5.51, m	125.9
13			140.2		140.3		139.9
14			42.8		42.8		43.0
15	*β*	1.83	29.7	1.83	29.7	2.37, m	29.1
	*α*	1.03		1.02		1.21, m	
16	*α*	2.60, m	26.7	2.61, m	26.7	2.11	25.4
	*β*	1.53, m		1.54		1.96	
17			48.8		49.0		48.5
18		2.54, s	55.2	2.54, s	55.2	2.67, m	54.0
19			73.8		73.8	1.45	39.9
20		1.31	43.3	1.33	43.2	1.04	39.9
21	*α*	1.73	27.4	1.73	27.4	1.40	31.6
	*β*	1.27		1.23		1.48	
22	*β*	1.74	39.2	1.75	39.2	2.00	38.0
	*α*	1.64		1.65		2.00	
23		0.91, s	28.7	0.92, s	28.7	1.13, s	28.9
24		1.00, s	22.4	1.01, s	22.3	0.96, s	21.9
25		1.04, s	17.0	1.07, s	17.0	1.00, s	17.1
26		0.81, s	17.7	0.84, s	17.7	1.08, s	18.0
27		1.36, s	25.1	1.44, s	25.1	1.25, s	24.5
28			182.4		182.4		180.6
29		1.23, s	27.2	1.23, s	27.2	1.00	22.5
30		0.95, d (6.7)	16.8	0.96, d (6.6)	16.8	1.00	18.0
1’			168.6		169.5		168.4
2’		5.87, d (13.0)	117.5	6.40, d (15.9)	116.0	6.78, d (15.9)	116.6
3’		6.88, d (13.0)	144.8	7.63, d (15.9)	146.4	8.03, d (15.9)	145.5
4’			127.9		127.4		126.7
5’		7.66, d (8.7)	133.8	7.47, d (8.6)	131.3	7.52, d (8.6)	131.1
6’		6.75, d (8.7)	116.0	6.82, d (8.6)	117.0	7.15, d (8.6)	117.2
7’			160.0		161.3		161.8
8’		6.75, d (8.7)	116.0	6.82, d (8.6)	117.0	7.15, d (8.6)	117.2
9’		7.66, d (8.7)	133.8	7.47, d (8.6)	131.3	7.52, d (8.6)	131.1

*^a^* Overlapped signals were reported without designating multiplicity. *^b^* NMR data were recorded in CD_3_OD. *^c^* NMR data were recorded in C_5_D_5_N.

**Table 2:** ^1^H (400 MHz) and ^13^C NMR (100 MHz) data of **4**–**6** (C_5_D_5_N, *δ* in ppm, *J* in Hz) *^a^*.

No.		4		5		6	
		*δ*_H_	*δ*_C_	*δ*_H_	*δ*_C_	*δ*_H_	*δ*_C_
1	*β*	2.33	49.0	2.33	49.1	2.03	44.1
	*α*	1.42, m		1.41, m		1.86, m	
2		4.32, ddd (10.8, 4.3, 3.7)	66.8	4.32, ddd (10.8, 4.3, 3.7)	66.9	4.49, dt (10.8, 4.5)	65.1
3		5.28, d (10.8)	85.5	5.29, d (10.8)	85.5	5.64, br s	81.4
4			40.4		40.3		39.2
5		1.10, m	56.0	1.11, m	56.1	1.53	50.9
6	*α*	1.51	19.1	1.53	19.1	1.47	18.7
	*β*	1.37		1.38		1.33	
7	*α*	1.57	33.7	1.56	33.8	1.62, m	33.7
	*β*	1.35		1.37		1.39	
8			40.3		40.5		40.6
9		1.74, m	48.4	1.76, m	48.5	1.95	48.5
10			38.7		38.8		39.2
11	*α*	1.98	24.2	1.99	24.2	2.06	24.1
	*β*	1.98		1.27		1.27	
12		5.47, m	126.2	5.48, m	126.2	5.49, m	126.3
13			139.5		139.5		139.5
14			43.1		43.1		43.0
15	*β*	2.31	29.1	2.32	29.1	2.33	29.0
	*α*	1.24, m		1.25, m		1.22, m	
16	*α*	2.30	25.3	2.32	25.4	2.30	25.3
	*β*	2.12		2.10		2.09	
17			48.7		48.8		48.7
18		2.77, d (11.8)	56.0	2.78, d (11.8)	56.1	2.76, d (11.6)	56.0
19		2.48, m	38.2	2.48, m	38.2	2.44, m	38.2
20			154.2		154.3		154.2
21	*α*	2.27	33.2	2.30	33.2	2.28	33.2
	*β*	2.43, m		2.45, m		2.40	
22	*β*	2.14	40.1	2.15	40.2	2.12, m	40.1
	*α*	2.03		2.02		2.02	
23		1.09, s	29.5	1.10, s	29.5	1.14, s	28.9
24		1.06, s	18.7	1.05, s	18.8	0.96, s	22.5
25		1.00, s	17.4	1.01, s	17.4	0.98, s	17.1
26		1.03, s	17.9	1.04, s	17.9	1.05, s	17.9
27		1.22, s	24.2	1.23, s	24.3	1.17, s	24.2
28			179.8		179.9		179.8
29		1.13, d (6.4)	17.1	1.14, d (6.1)	17.1	1.11, d (6.1)	17.1
30	a	4.84, br s	105.6	4.84, br s	105.6	4.83, br s	105.6
	b	4.79, br s		4.79, br s		4.79, br s	
1′			168.4		168.4		168.4
2′		6.70, d (15.9)	116.6	6.75, d (15.8)	116.7	6.89, d (15.9)	116.7
3′		8.02, d (15.9)	145.3	8.04, d (15.8)	145.6	8.07, d (15.9)	146.0
4′			126.7		127.2		127.1
5′		7.57, d (8.5)	131.1	7.31, m	112.0	7.24, m	111.8
6′		7.18, d (8.5)	117.3		149.5		149.4
7′			161.8		151.5		151.5
8′		7.18, d (8.5)	117.3	7.23, m	117.3	7.20, m	117.2
9′		7.57, d (8.5)	131.1	7.23, m	124.1	7.20, m	124.5
Ome				3.80, s	56.4	3.72, s	56.3

*^a^* Overlapped signals were reported without designating multiplicity.

**Table 3:** PTP1B inhibitory activity.

Compounds	IC_50_ (μM)	Compounds	IC_50_ (μM)
4	48.67 ± 5.17	11	26.20 ± 1.31
5	23.74 ± 0.73	12	16.20 ± 0.57
6	34.01 ± 4.88	13	10.32 ± 1.21
10	19.15 ± 0.22	15	17.12 ± 1.67
Oleanolic acid *^a^*	10.19 ± 0.12		

*^a^* Positive control.

## Data Availability

The data presented in this study are available in this article.

## Supplementary Materials

The following supporting information can be downloaded at: https://www.mdpi.com/article/10.3390/molecules29071640/s1, Figures S1–S54: 1D, 2D NMR, HRESIMS, IR, and UV spectra of compounds **1**–**6**.
