# Supplementary material for: Triterpenoids from the Leaves of Diospyros digyna and Their PTP1B Inhibitory Activity
Source: Molecules. 2024 Apr 5;29(7):1640. doi: 10.3390/molecules29071640 (PMC11013491; doi:10.3390/molecules29071640)
Supplement: Supplementary file 1 [file molecules-29-01640-s001.zip › molecules-2939492-supplementary.pdf]

Supplementary Materials

Triterpenoids from the leaves of *Diospyros digyna* and their PTP1B inhibitory activity

Lan Huang [1,2,†], Ziqi Wang [1,2,3,†], Fangxin Wang [1,2], Song Wang [1,2], Dezhi Wang [1,2], Meihua Gao [3], Hua Li [3], Min Song [1,2] and Xiaoqi Zhang [1,2,*]

[1] Guangdong Provincial Engineering Research Center for Modernization of TCM, Jinan University, Guangzhou 510632, P. R. China; huanglan@stu2021.jnu.edu.cn (L.H.); wangziqi@stu2021.jnu.edu.cn (Z.W.); wfxwfx@stu2022.jnu.edu.cn (F.W.); wangsong@stu2022.jnu.edu.cn (S.W.); dezhiwoo125@stu2020.jnu.edu.cn (D.W.); songm1017@jnu.edu.cn (M.S.)
[2] NMPA Key Laboratory for Quality Evaluation of TCM, Jinan University, Guangzhou 510632, P. R. China
[3] Guangdong Institute for Drug Control, Guangzhou 510663, P. R. China; gaomeihua@gdidc.org.cn (M.G.); bgs@gdidc.org.cn (H.L.)
* Correspondence: tzhxq01@jnu.edu.cn
† These authors contributed equally to this work.

Content

Figure S1. ^{1}H NMR spectrum of **1** (400 MHz, CD_3OD)....4
Figure S2. ^{13}C NMR spectrum of **1** (100 MHz, CD_3OD)....4
Figure S3. HSQC spectrum of **1** in CD_3OD....5
Figure S4. HMBC spectrum of **1** in CD_3OD....5
Figure S5. ^{1}H-^{1}H COSY spectrum of **1** in CD_3OD....6
Figure S6. NOESY spectrum of **1** in CD_3OD....6
Figure S7. (+)-HRESIMS spectrum of **1**....7
Figure S8. IR spectrum of **1**....7
Figure S9. UV spectrum of **1**....7
Figure S10. ^{1}H NMR spectrum of **2** (400 MHz, CD_3OD)....8
Figure S11. ^{13}C NMR spectrum of **2** (100 MHz, CD_3OD)....8
Figure S12. HSQC spectrum of **2** in CD_3OD....9
Figure S13. HMBC spectrum of **2** in CD_3OD....9
Figure S14. ^{1}H-^{1}H COSY spectrum of **2** in CD_3OD....10
Figure S15. NOESY spectrum of **2** in CD_3OD....10
Figure S16. (+)-HRESIMS spectrum of **2**....11
Figure S17. IR spectrum of **2**....11
Figure S18. UV spectrum of **2**....11
Figure S19. ^{1}H NMR spectrum of **3** (400 MHz, C_5D_5N)....12
Figure S20. ^{13}C NMR spectrum of **3** (100 MHz, C_5D_5N)....12
Figure S21. HSQC spectrum of **3** in C_5D_5N....13
Figure S22. HMBC spectrum of **3** in C_5D_5N....13
Figure S23. ^{1}H-^{1}H COSY spectrum of **3** in C_5D_5N....14
Figure S24. NOESY spectrum of **3** in C_5D_5N....14
Figure S25. (+)-HRESIMS spectrum of **3**....15
Figure S26. IR spectrum of **3**....15
Figure S27. UV spectrum of **3**....15
Figure S28. ^{1}H NMR spectrum of **4** (400 MHz, C_5D_5N)....16
Figure S29. ^{13}C NMR spectrum of **4** (100 MHz, C_5D_5N)....16
Figure S30. HSQC spectrum of **4** in C_5D_5N....17
Figure S31. HMBC spectrum of **4** in C_5D_5N....17
Figure S32. ^{1}H-^{1}H COSY spectrum of **4** in C_5D_5N....18
Figure S33. NOESY spectrum of **4** in C_5D_5N....18
Figure S34. (+)-HRESIMS spectrum of **4**....19
Figure S35. IR spectrum of **4**....19
Figure S36. UV spectrum of **4**....19
Figure S37. ^{1}H NMR spectrum of **5** (400 MHz, C_5D_5N)....20
Figure S38. ^{13}C NMR spectrum of **5** (100 MHz, C_5D_5N)....20
Figure S39. HSQC spectrum of **5** in C_5D_5N....21
Figure S40. HMBC spectrum of **5** in C_5D_5N....21
Figure S41. ^{1}H-^{1}H COSY spectrum of **5** in C_5D_5N....22
Figure S42. NOESY spectrum of **5** in C_5D_5N....22
Figure S43. (+)-HRESIMS spectrum of **5**....23

Figure S44. IR spectrum of **5** 23
Figure S45. UV spectrum of **5** 23
Figure S46. ^{1}H NMR spectrum of **6** (400 MHz, C_5D_5N) 24
Figure S47. ^{13}C NMR spectrum of **6** (100 MHz, C_5D_5N) 24
Figure S48. HSQC spectrum of **6** in C_5D_5N 25
Figure S49. HMBC spectrum of **6** in C_5D_5N 25
Figure S50. ^{1}H-^{1}H COSY spectrum of **6** in C_5D_5N 26
Figure S51. NOESY spectrum of **6** in C_5D_5N 26
Figure S52. (+)-HRESIMS spectrum of **6** 27
Figure S53. IR spectrum of **6** 27
Figure S54. UV spectrum of **6** 27

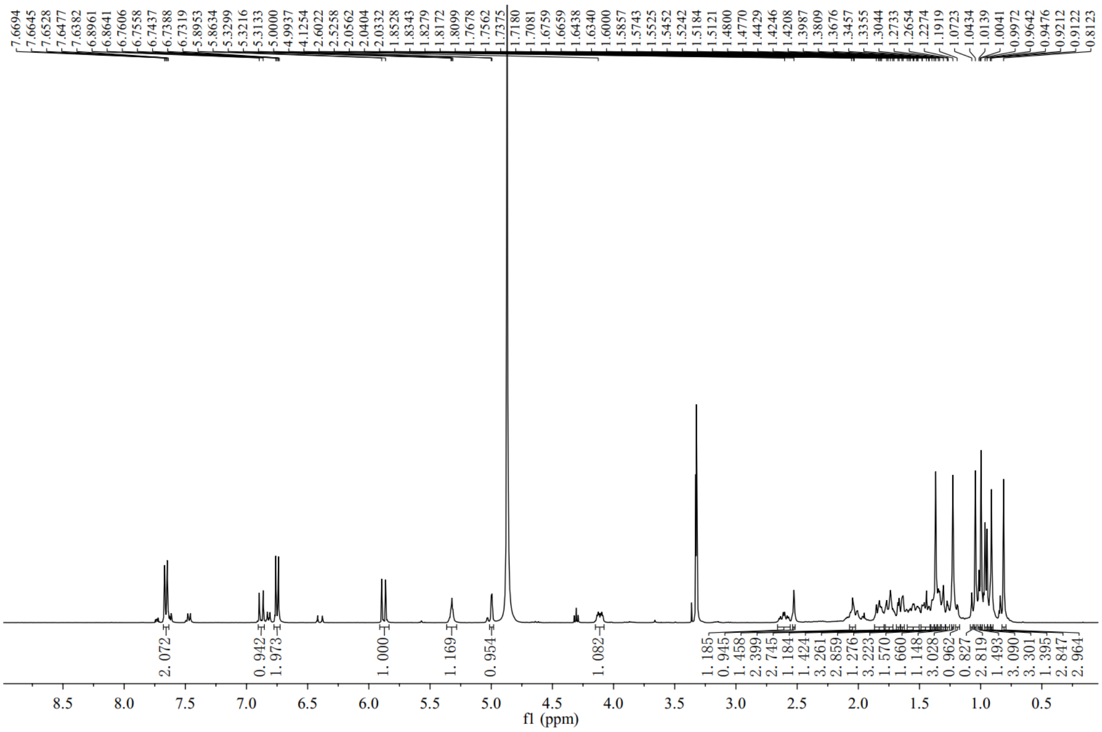

Figure S1. ^{1}H NMR spectrum of **1** (400 MHz, CD_3OD)

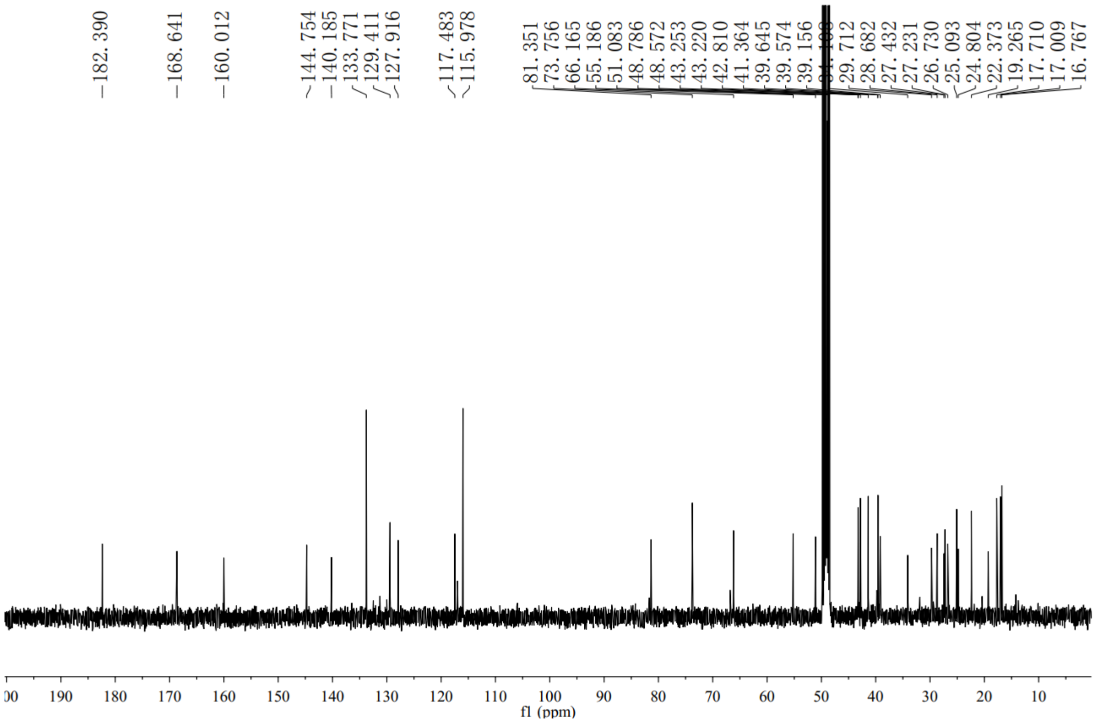

Figure S2. ^{13}C NMR spectrum of **1** (100 MHz, CD_3OD)

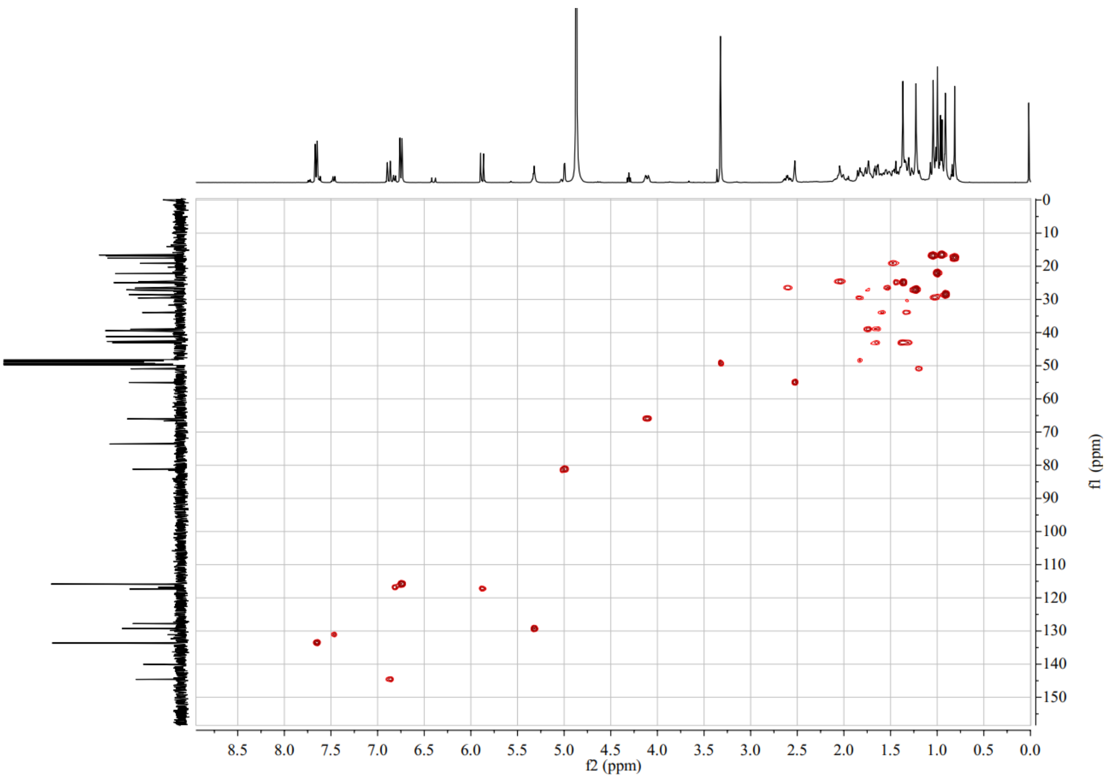

Figure S3. HSQC spectrum of **1** in CD_3OD

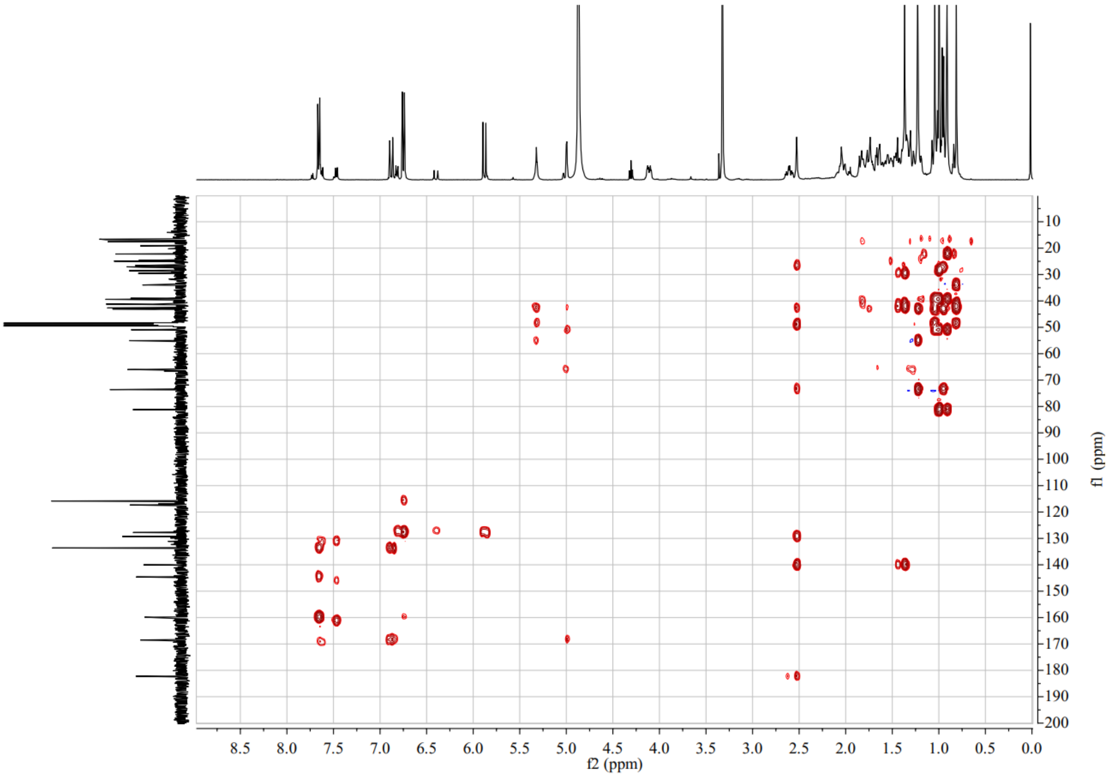

Figure S4. HMBC spectrum of **1** in CD_3OD

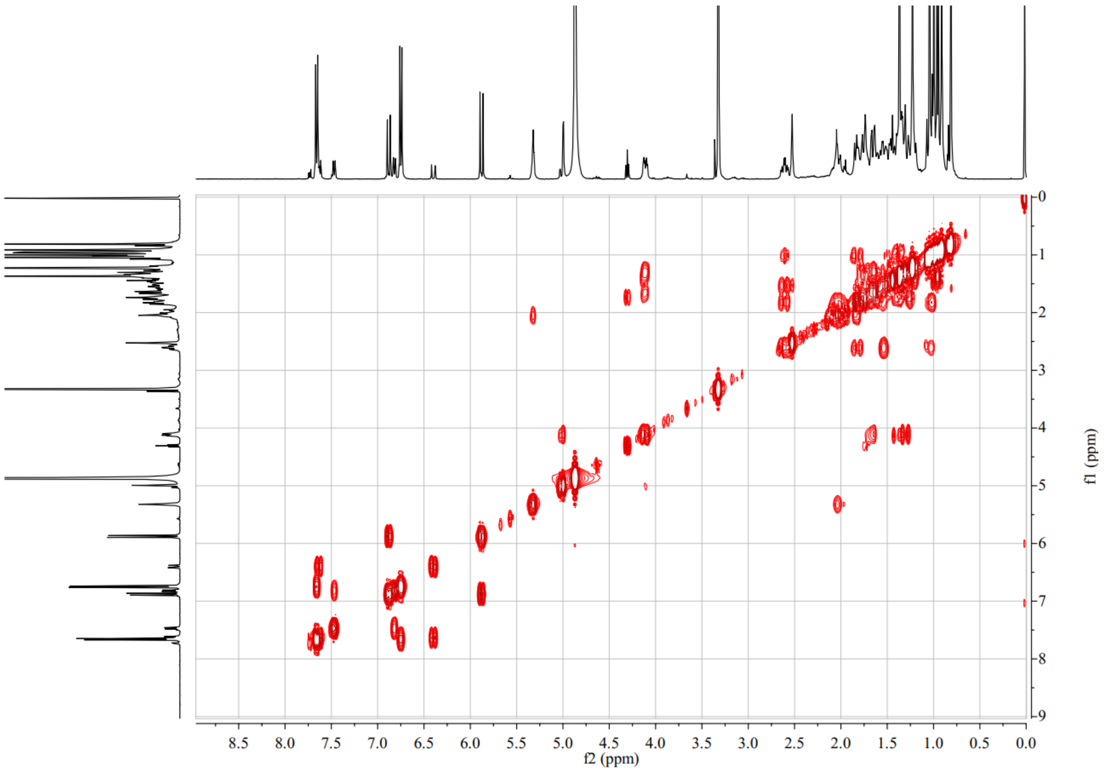

Figure S5. ^{1}H-^{1}H COSY spectrum of **1** in CD_3OD

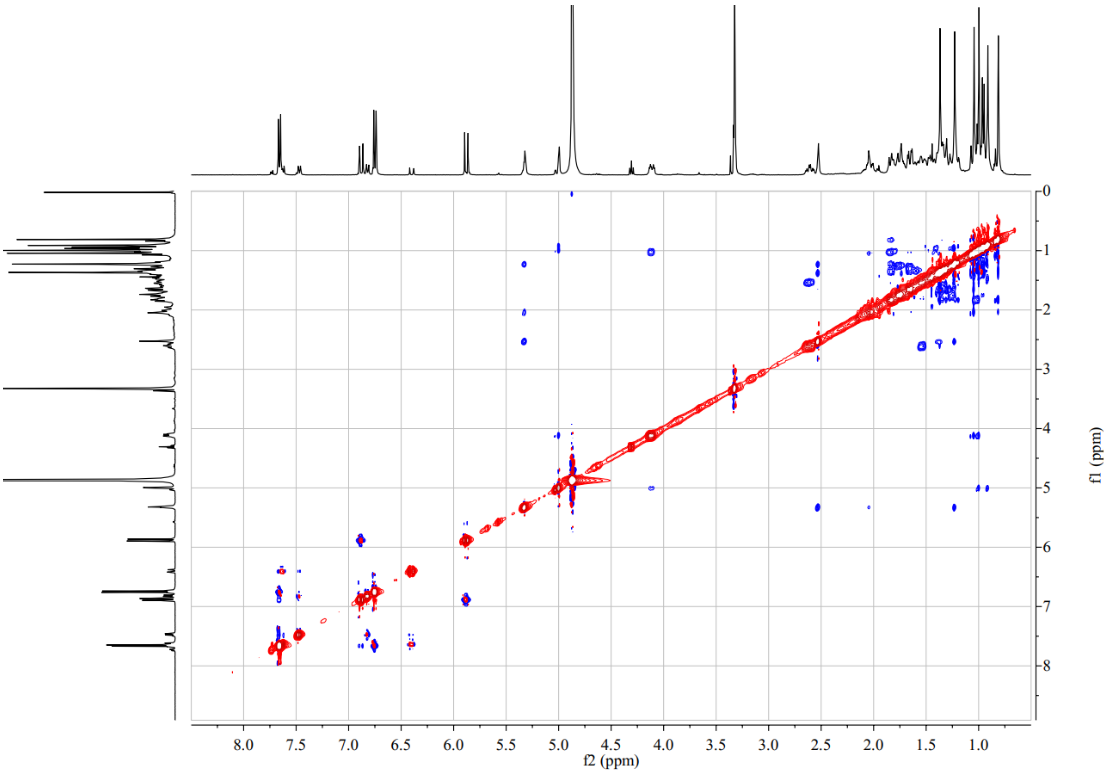

Figure S6. NOESY spectrum of **1** in CD_3OD

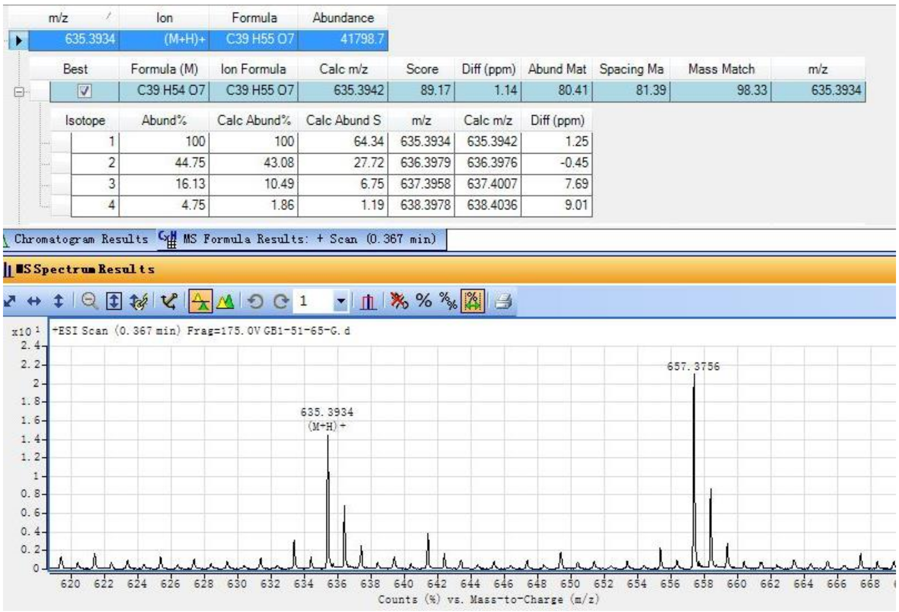

Figure S7. (+)-HRESIMS spectrum of **1**

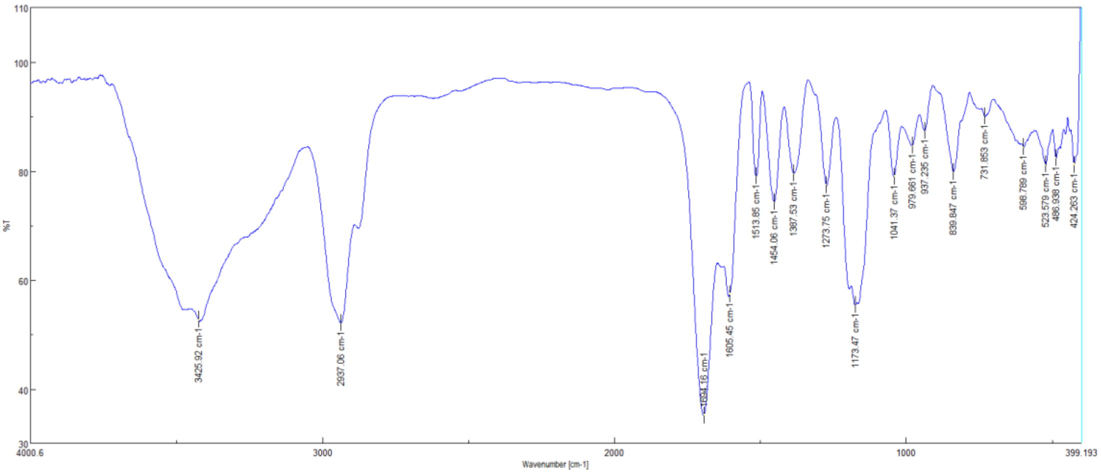

Figure S8. IR spectrum of **1**

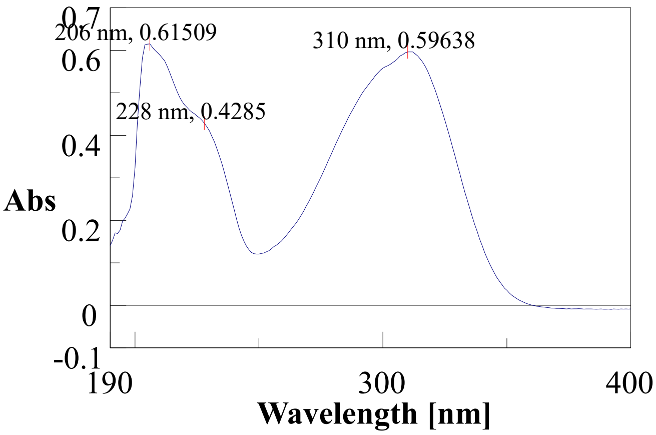

Figure S9. UV spectrum of **1**

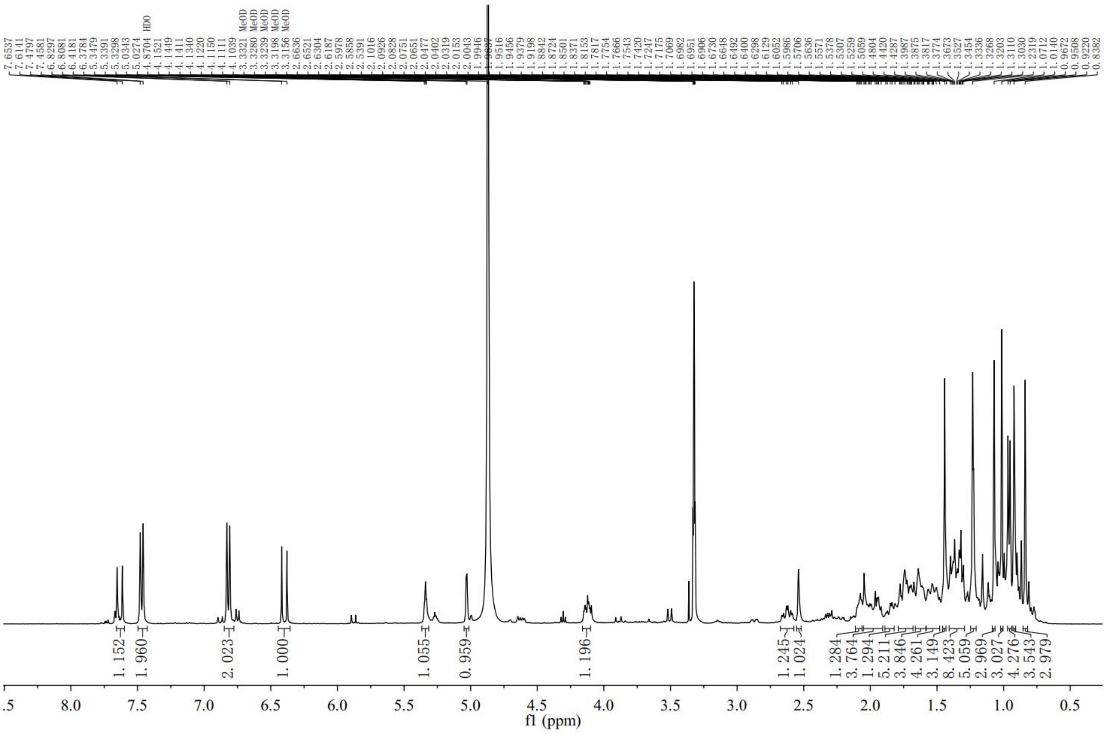

Figure S10. ^1H NMR spectrum of **2** (400 MHz, CD_3OD)

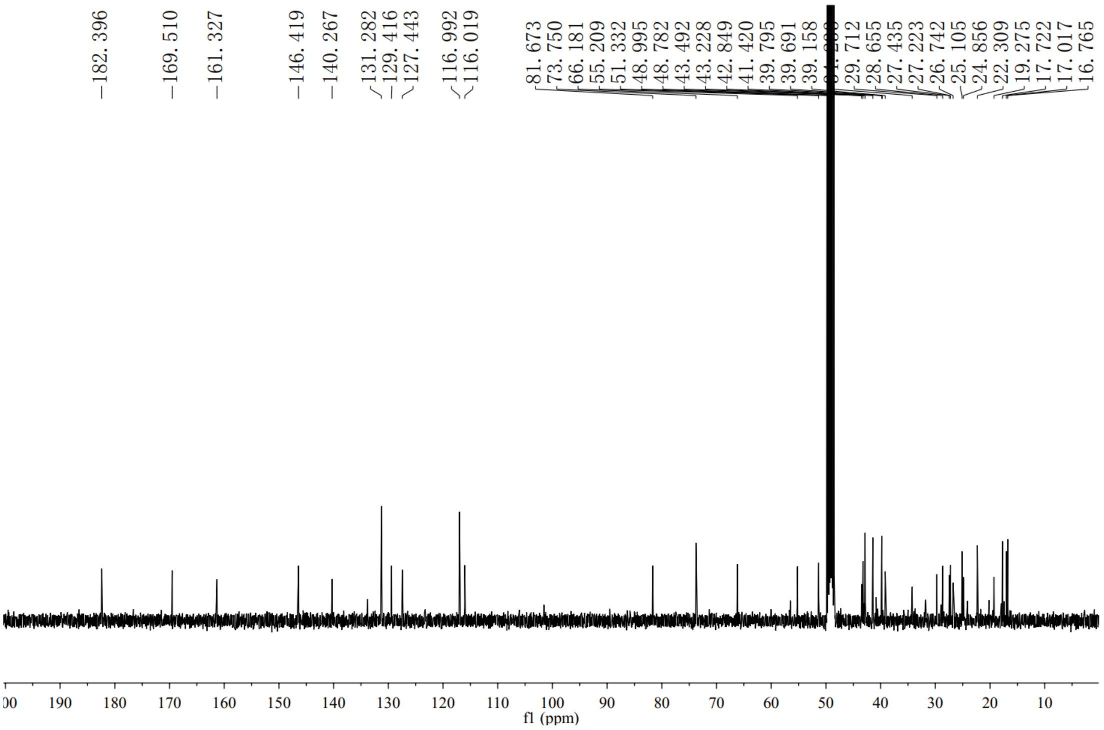

Figure S11. ^{13}C NMR spectrum of **2** (100 MHz, CD_3OD)

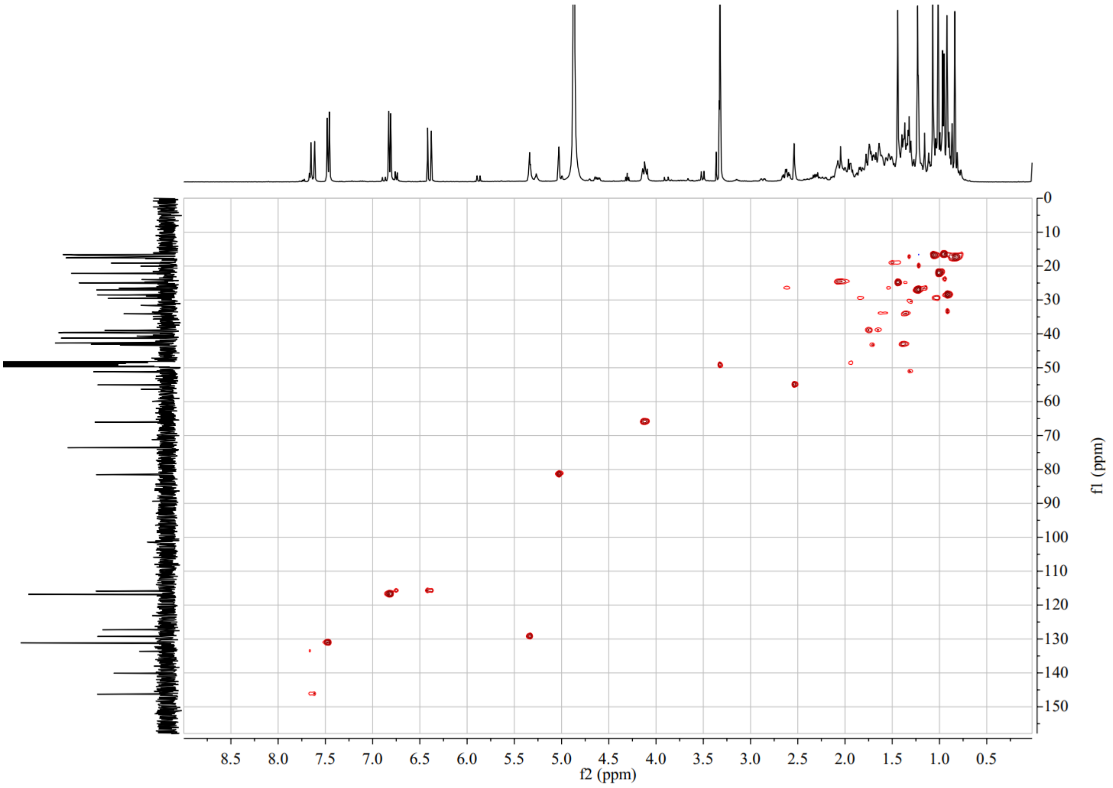

Figure S12. HSQC spectrum of **2** in CD3OD

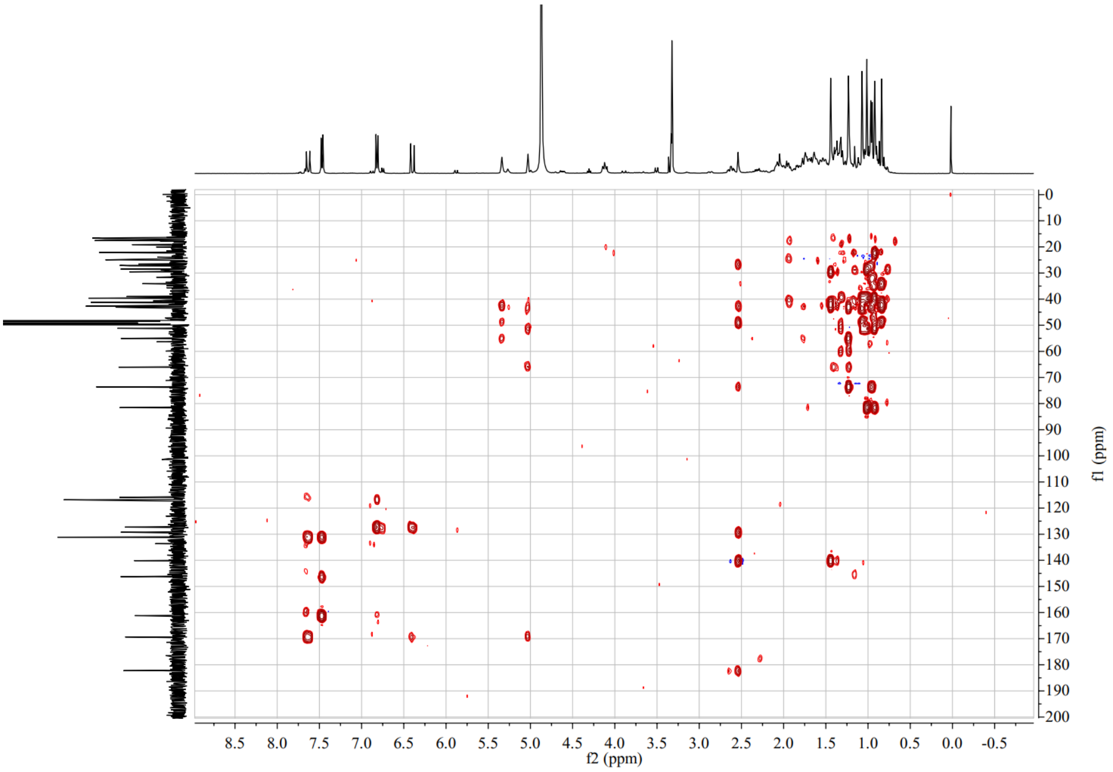

Figure S13. HMBC spectrum of **2** in CD3OD

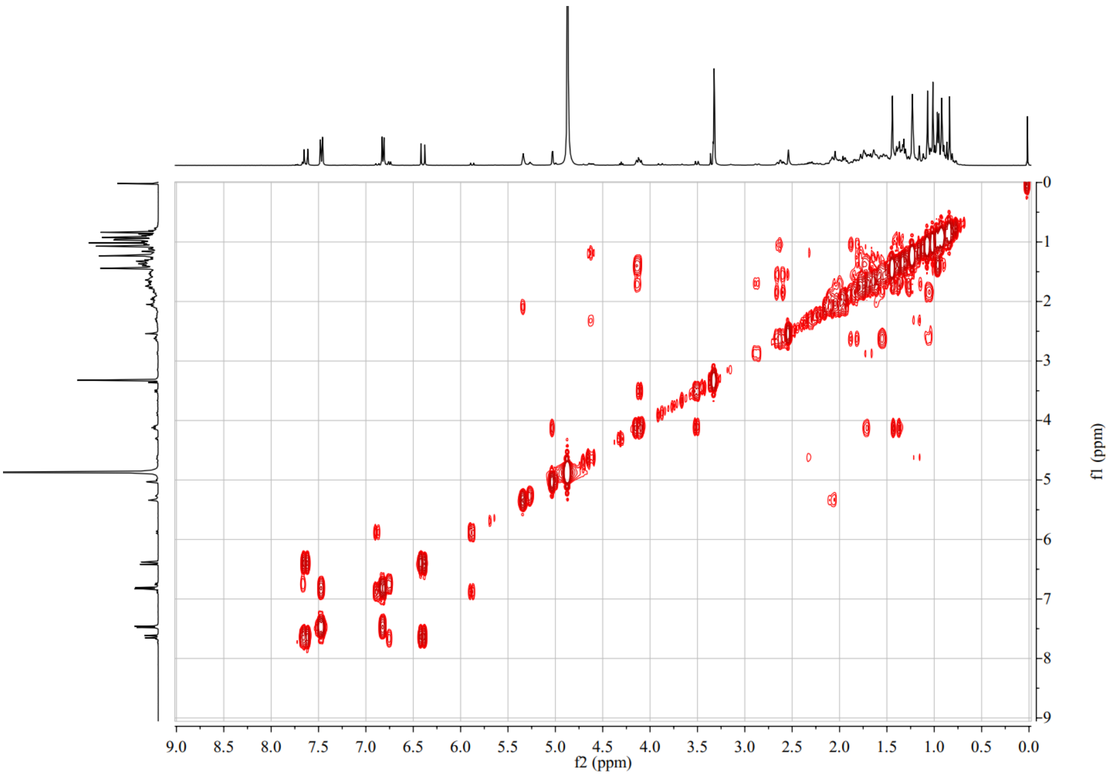

Figure S14. ^1H-^1H COSY spectrum of **2** in CD_3OD

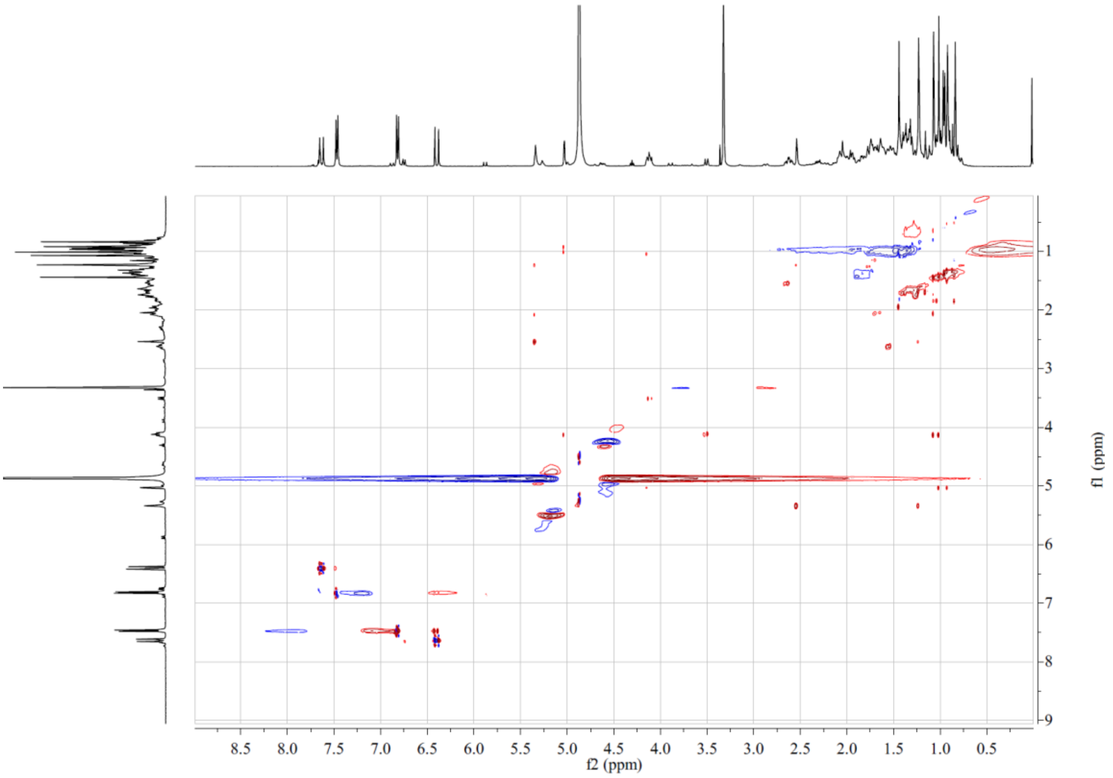

Figure S15. NOESY spectrum of **2** in CD_3OD

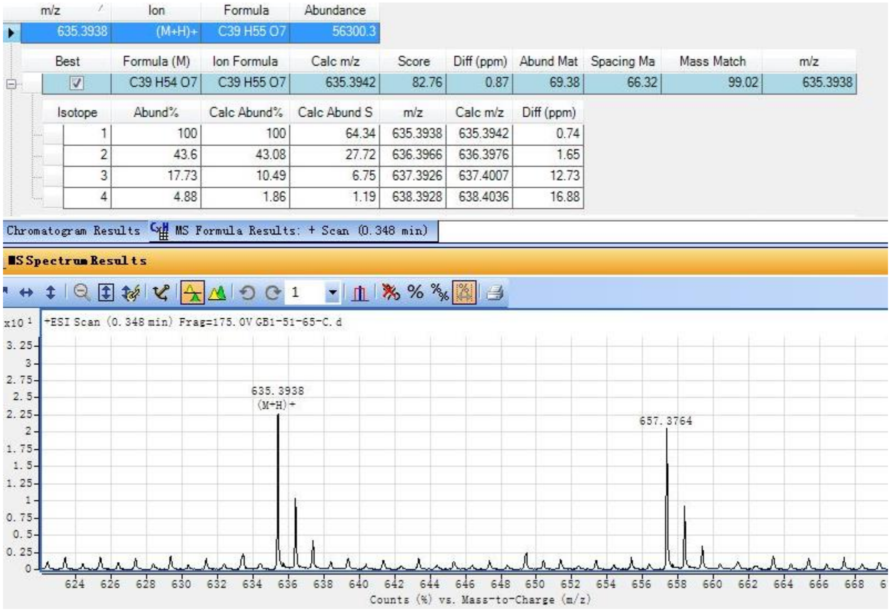

m/z	Ion	Formula	Abundance
635.3938	(M+H)+	C39 H55 O7	56300.3

Best	Formula (M)	Ion Formula	Calc m/z	Score	Diff (ppm)	Abund Mat	Spacing Ma	Mass Match	m/z
✓	C39 H54 O7	C39 H55 O7	635.3942	82.76	0.87	69.38	66.32	99.02	635.3938

Isotope	Abund%	Calc Abund%	Calc Abund S	m/z	Calc m/z	Diff (ppm)
1	100	100	64.34	635.3938	635.3942	0.74
2	43.6	43.08	27.72	636.3966	636.3976	1.65
3	17.73	10.49	6.75	637.3926	637.4007	12.73
4	4.88	1.86	1.19	638.3928	638.4036	16.88

Figure S16. (+)-HRESIMS spectrum of **2**

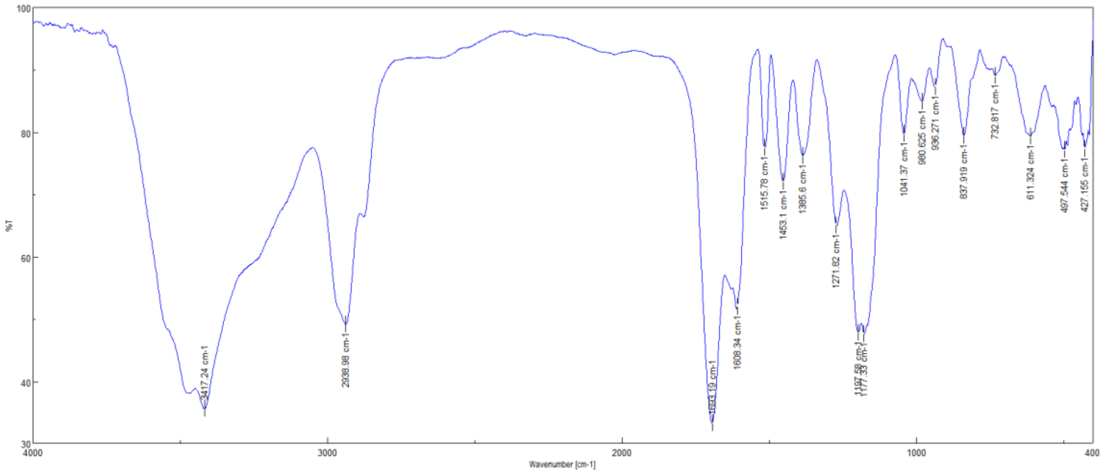

Figure S17. IR spectrum of **2**

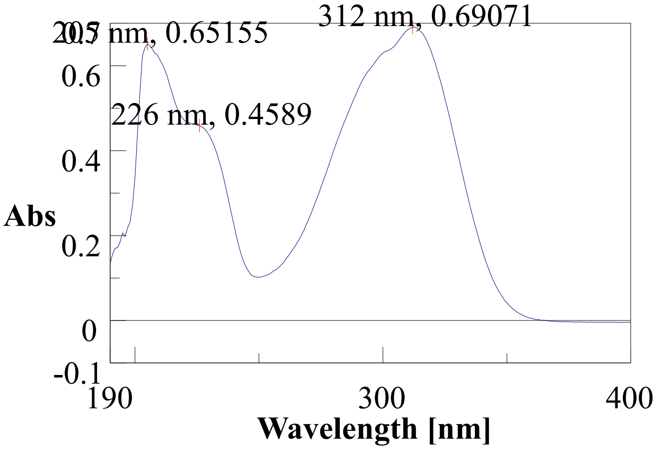

Figure S18. UV spectrum of **2**

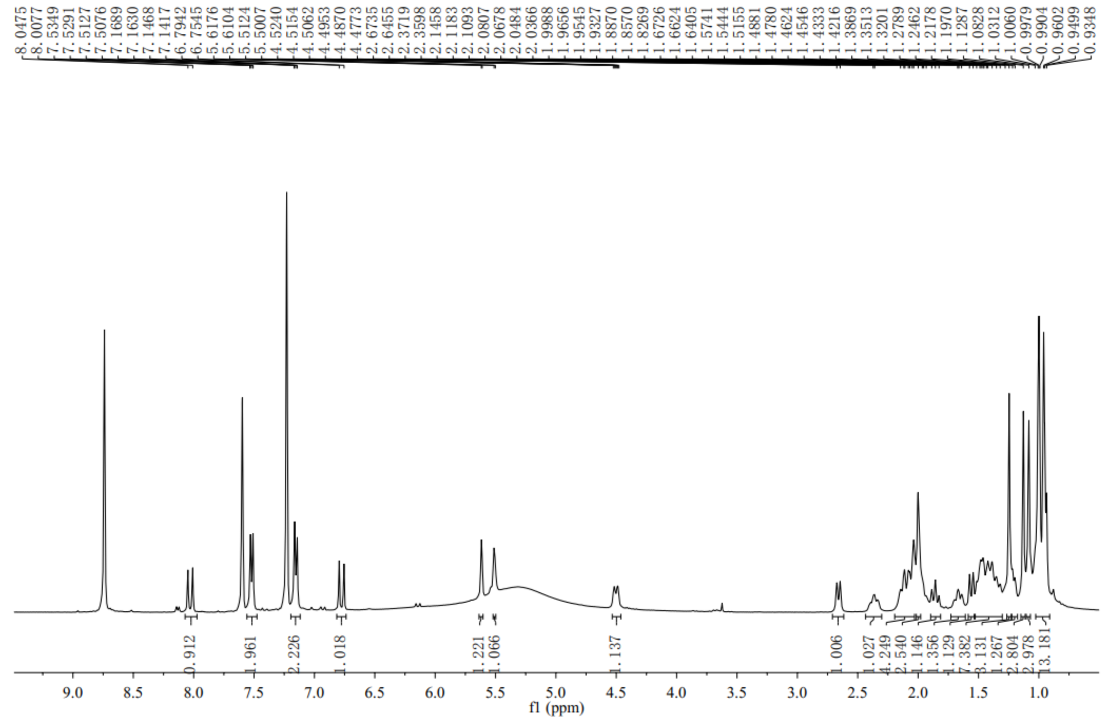

Figure S19. ^{1}H NMR spectrum of **3** (400 MHz, C_5D_5N)

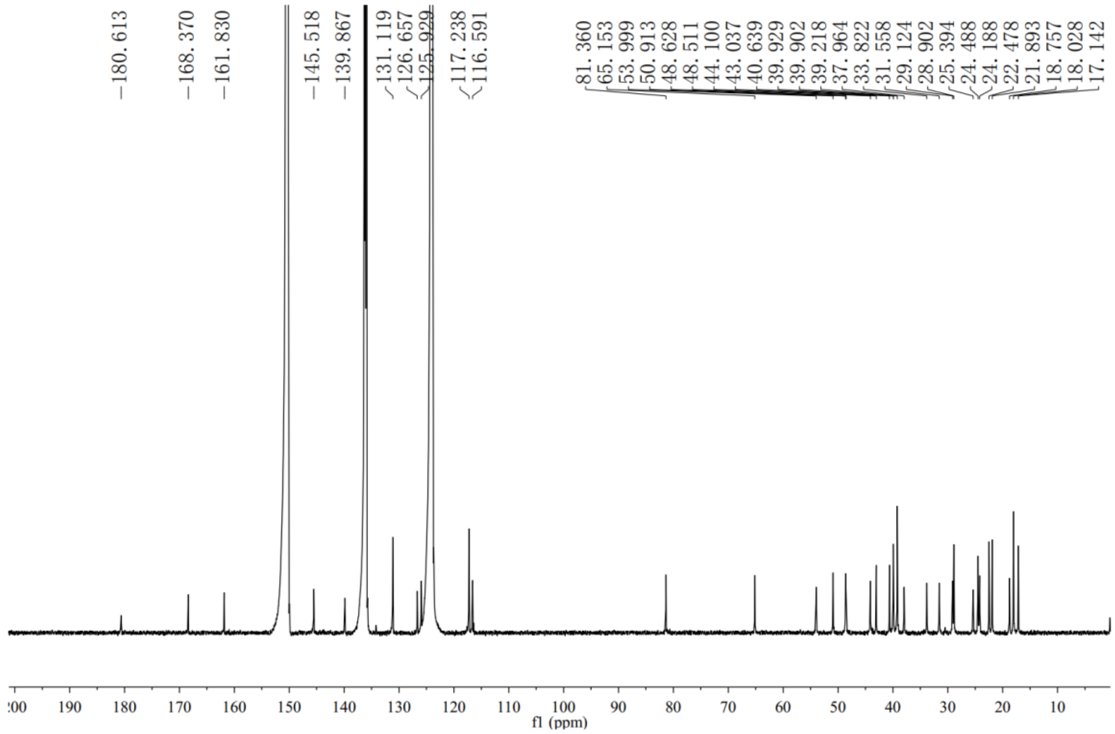

Figure S20. ^{13}C NMR spectrum of **3** (100 MHz, C_5D_5N)

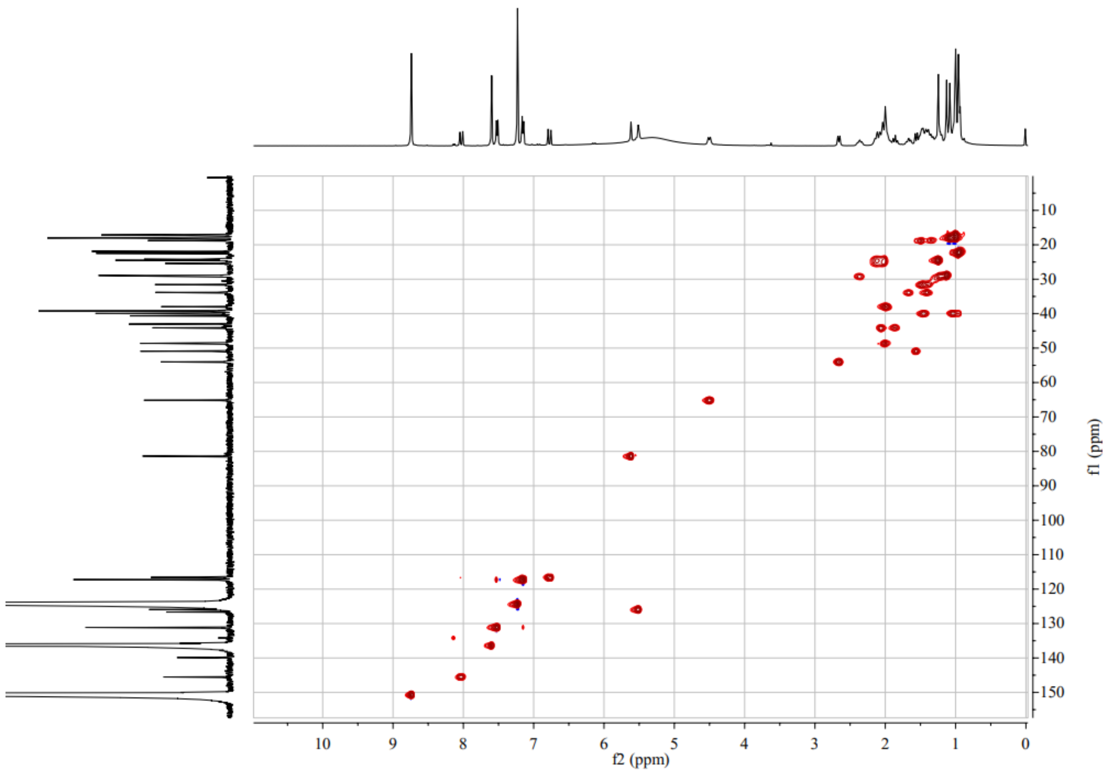

Figure S21. HSQC spectrum of **3** in C_5D_5N

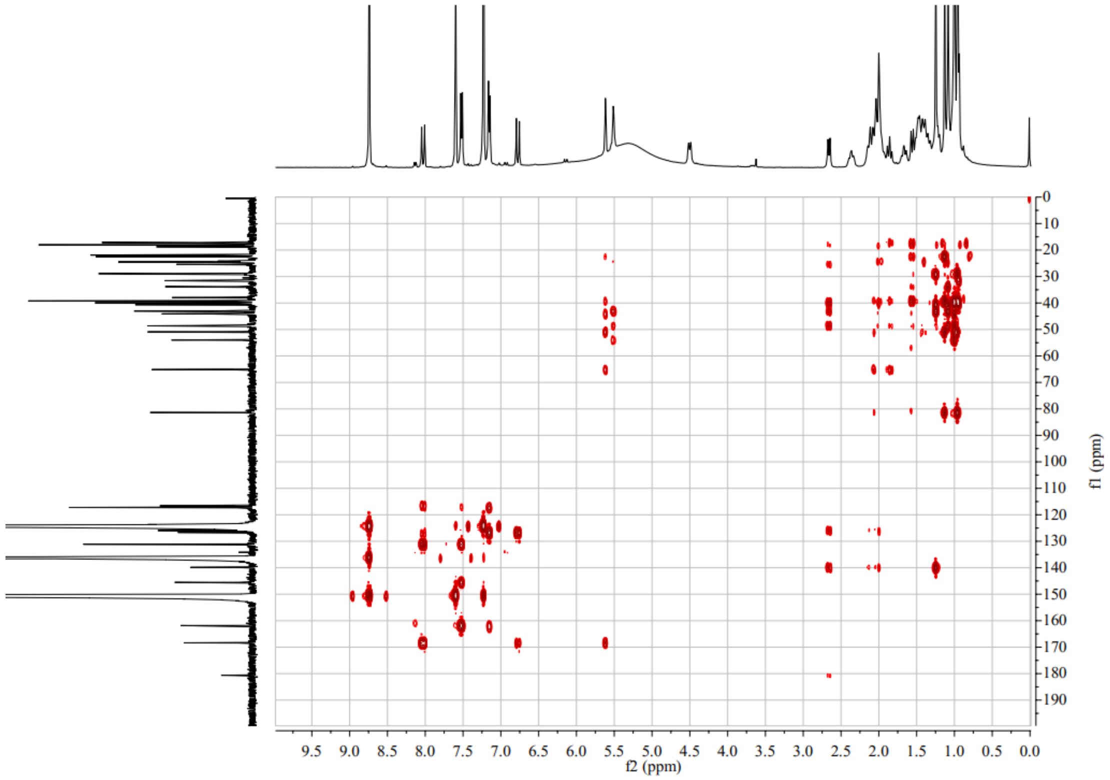

Figure S22. HMBC spectrum of **3** in C_5D_5N

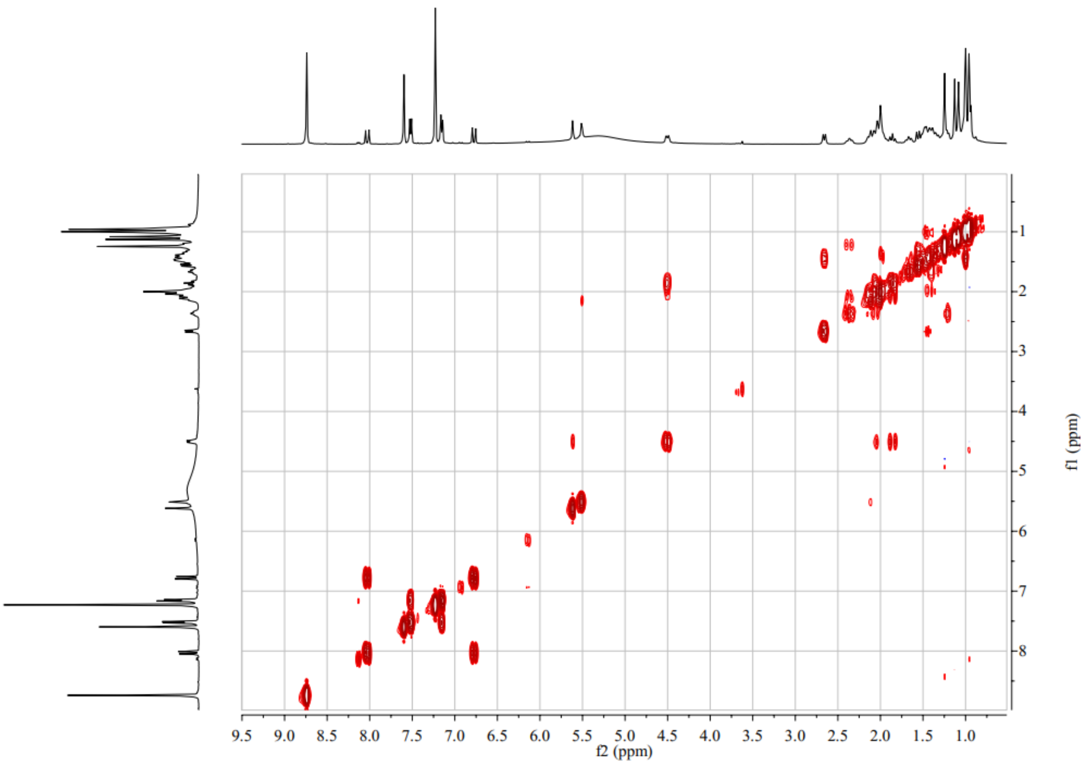

Figure S23. ^1H-^1H COSY spectrum of **3** in C_5D_5N

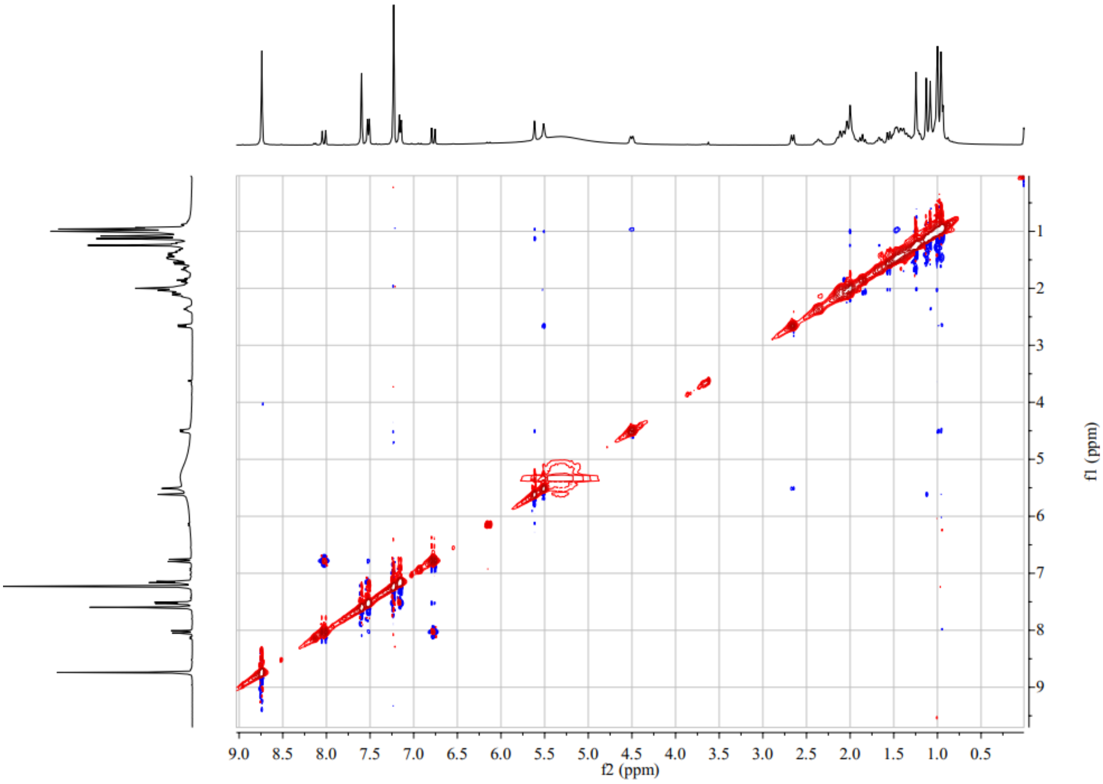

Figure S24. NOESY spectrum of **3** in C_5D_5N

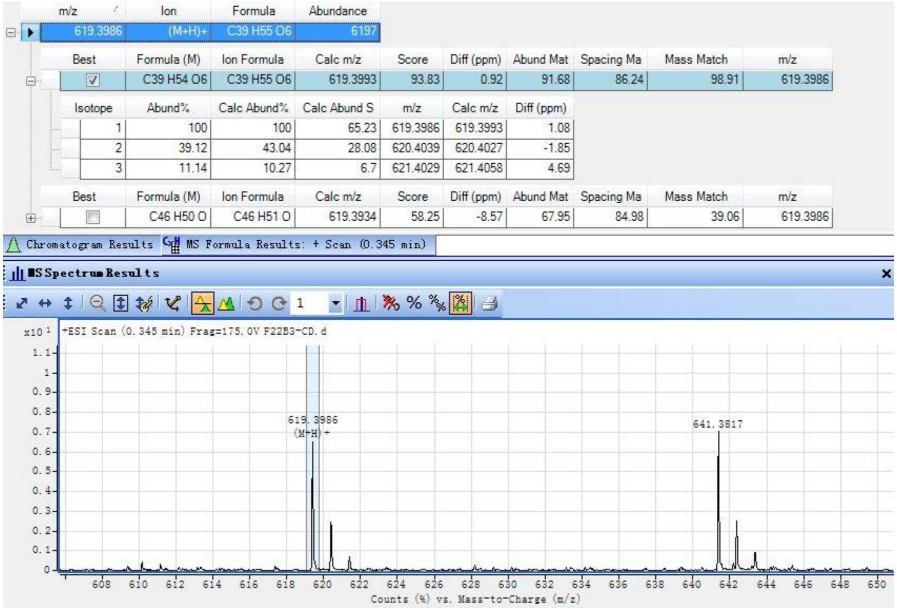

Figure S25. (+)-HRESIMS spectrum of **3**

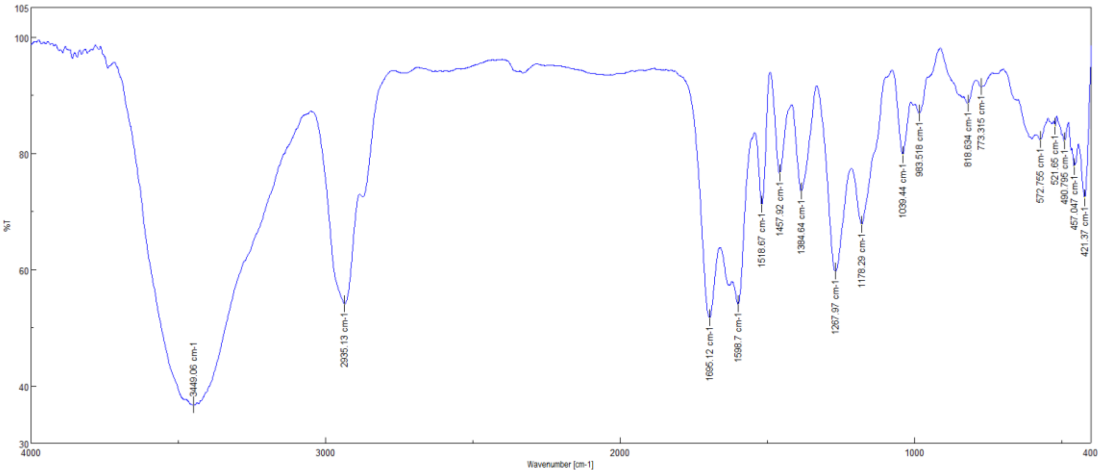

Figure S26. IR spectrum of **3**

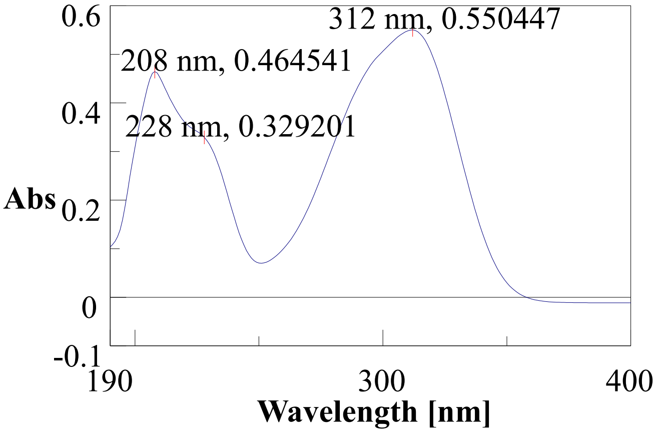

Figure S27. UV spectrum of **3**

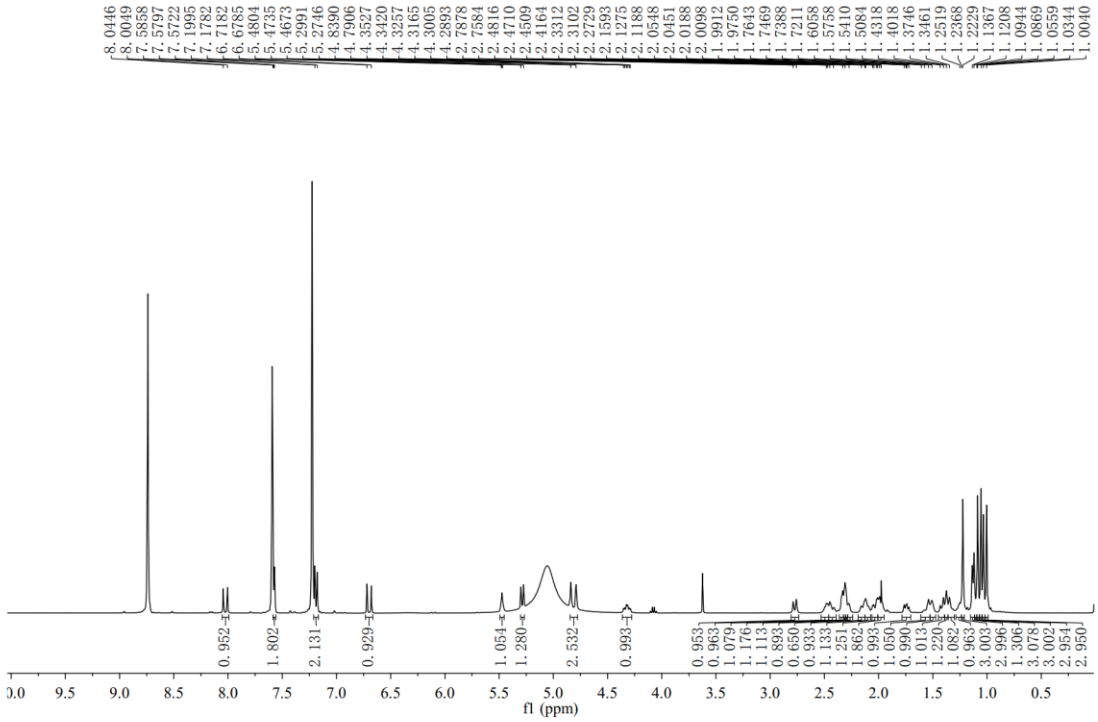

Figure S28. ^{1}H NMR spectrum of **4** (400 MHz, C_5D_5N)

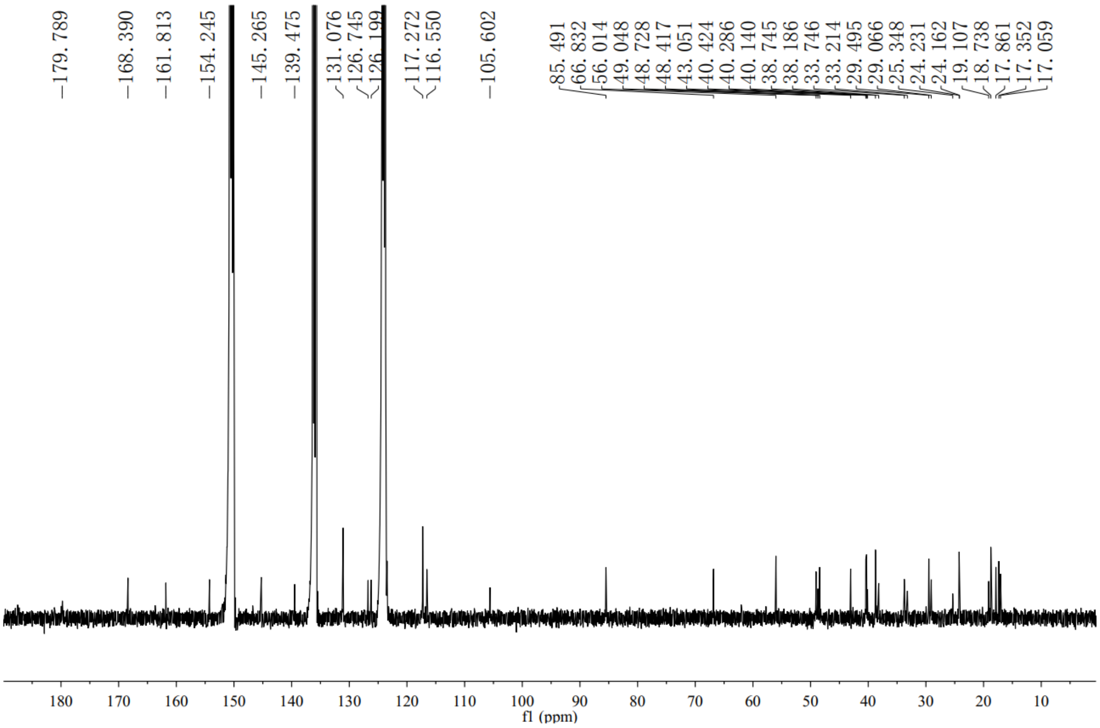

Figure S29. ^{13}C NMR spectrum of **4** (100 MHz, C_5D_5N)

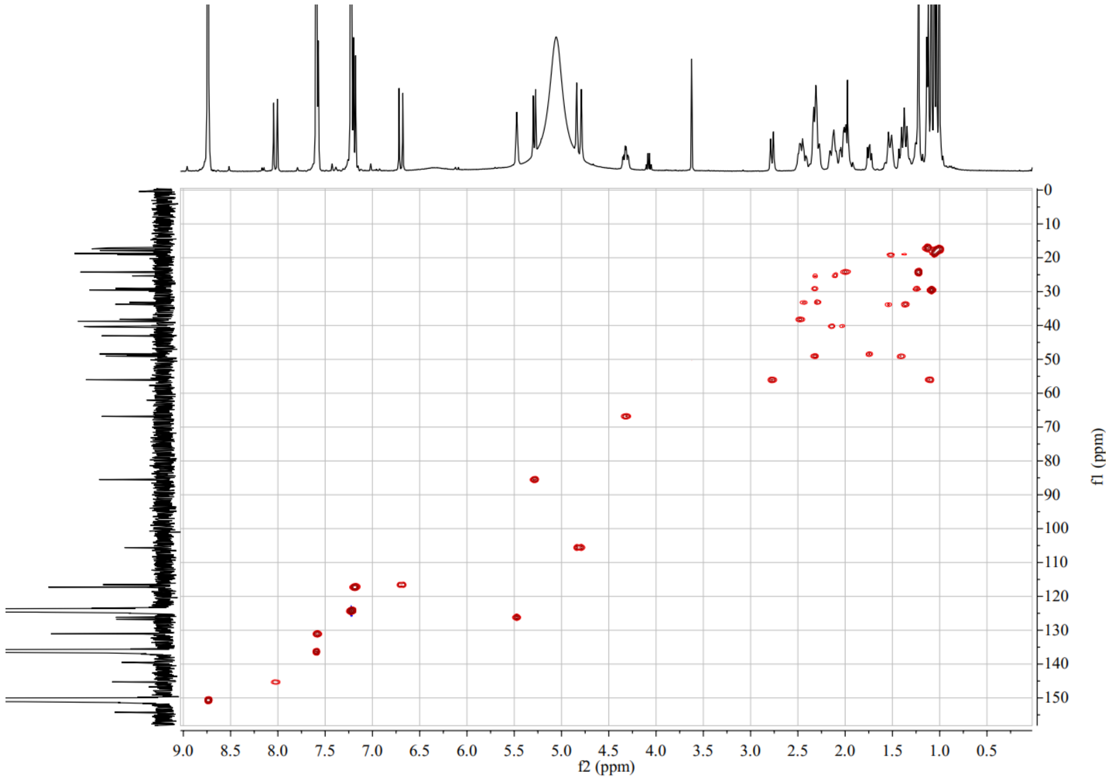

Figure S30. HSQC spectrum of **4** in C_5D_5N

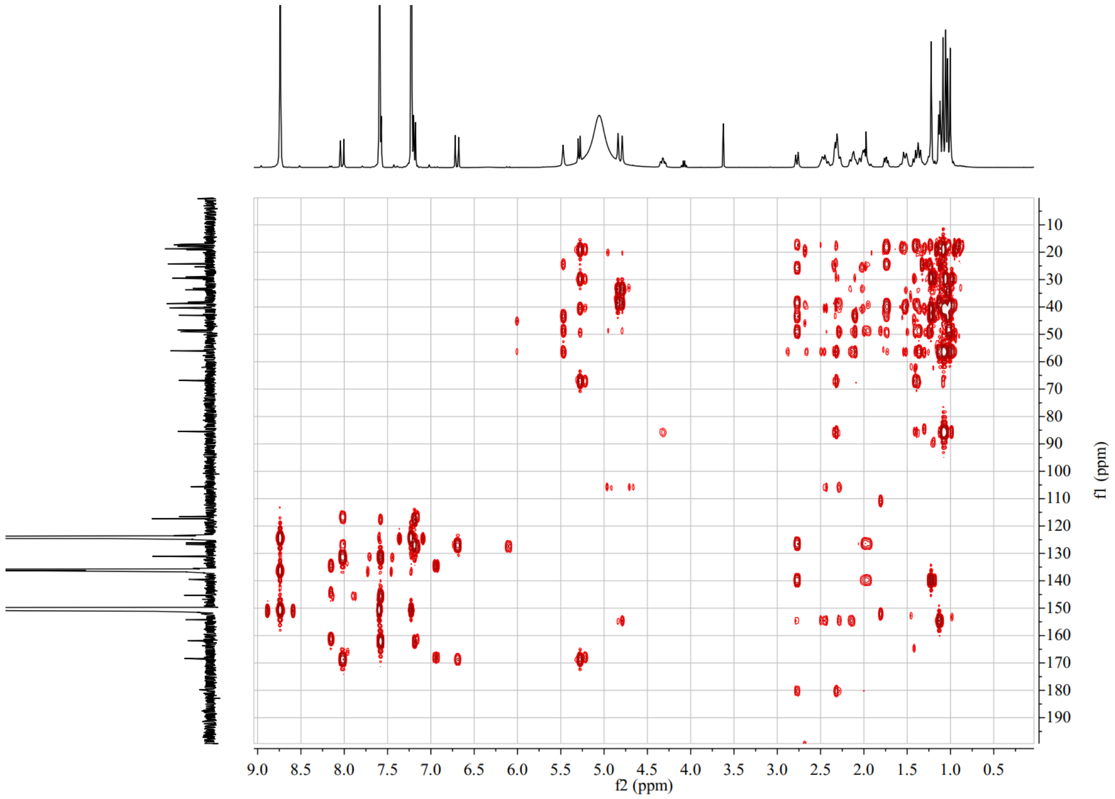

Figure S31. HMBC spectrum of **4** in C_5D_5N

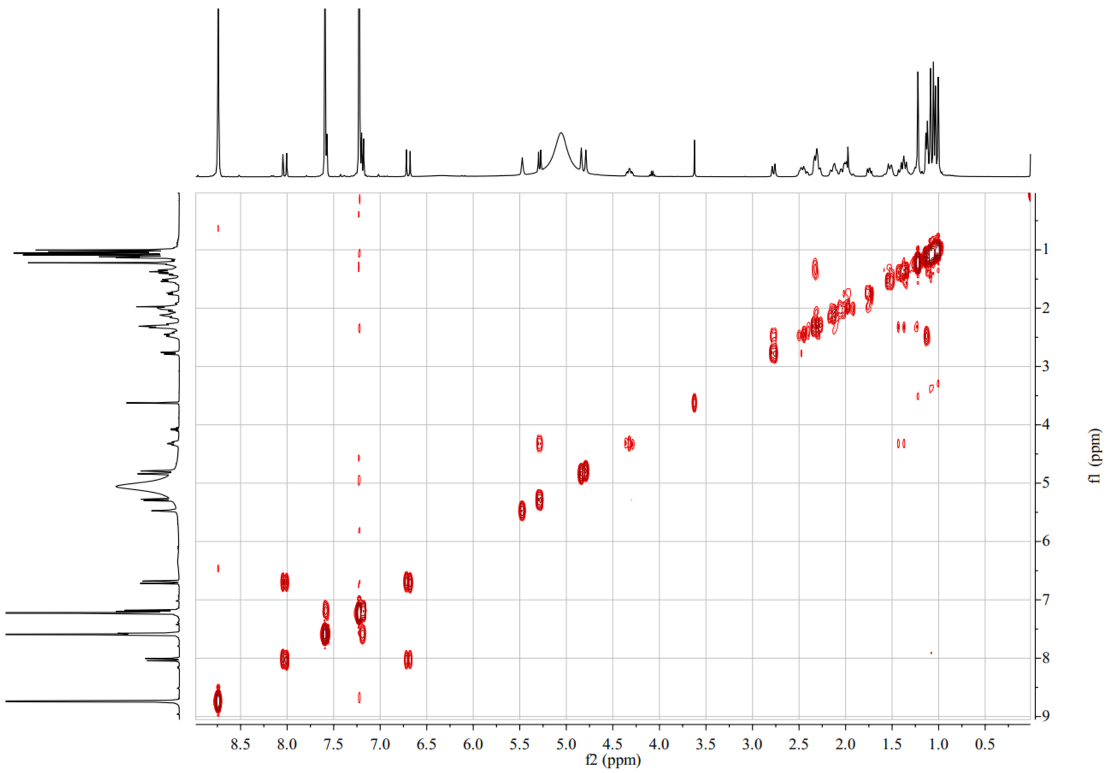

Figure S32. ^1H-^1H COSY spectrum of **4** in C_5D_5N

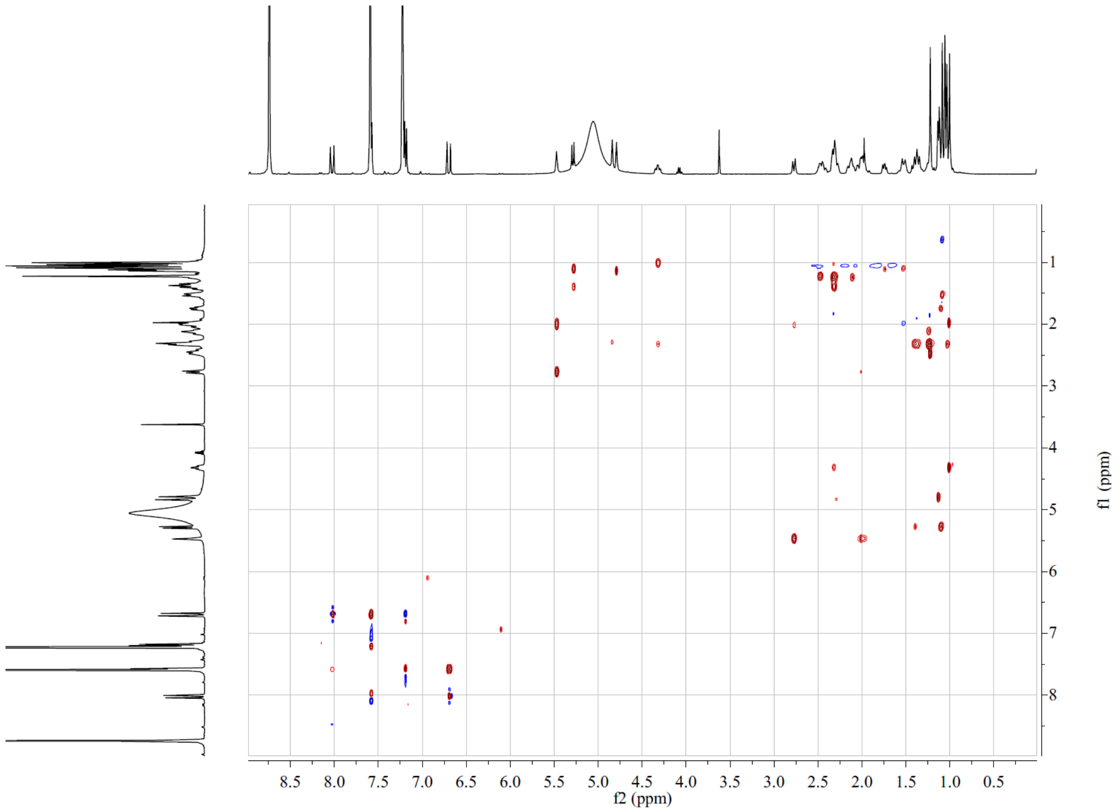

Figure S33. NOESY spectrum of **4** in C_5D_5N

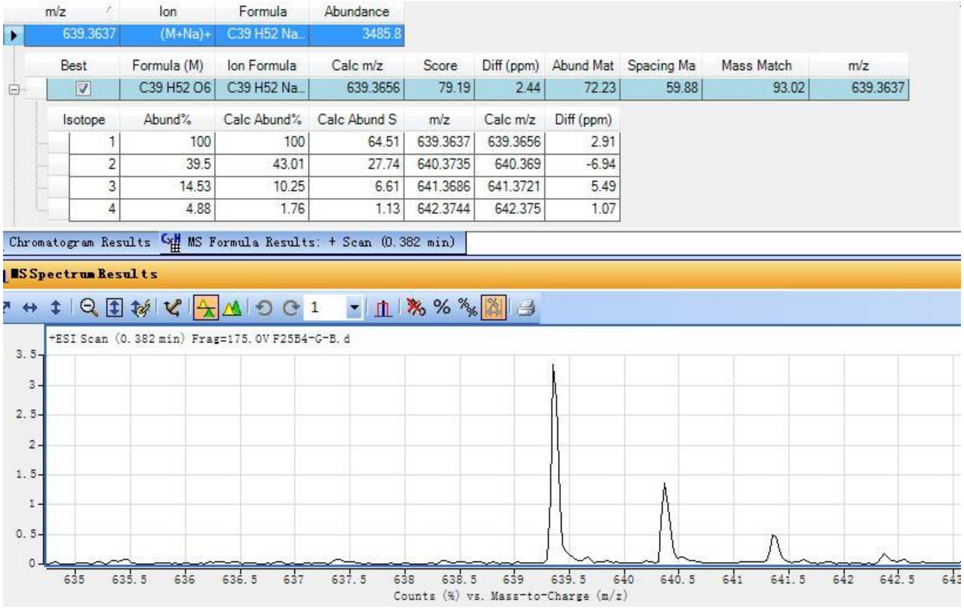

Figure S34. (+)-HRESIMS spectrum of **4**

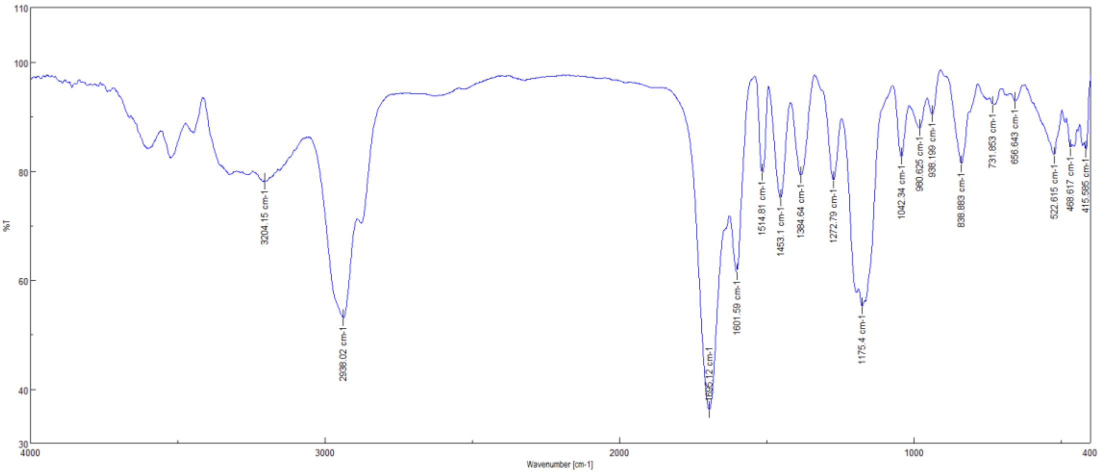

Figure S35. IR spectrum of **4**

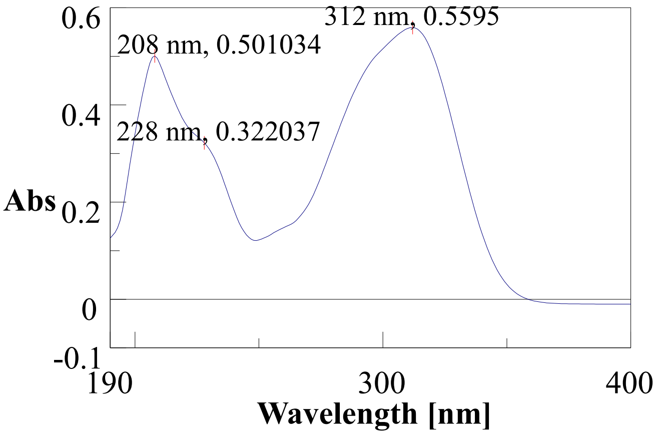

Figure S36. UV spectrum of **4**

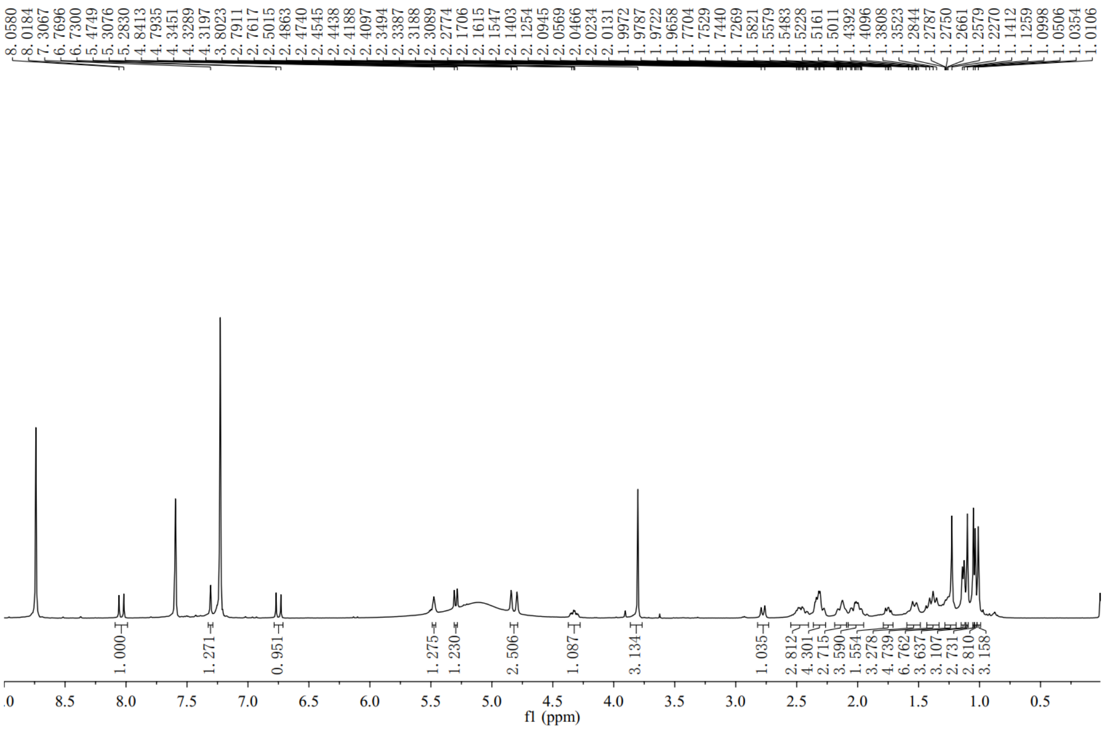

Figure S37. ^{1}H NMR spectrum of **5** (400 MHz, C_5D_5N)

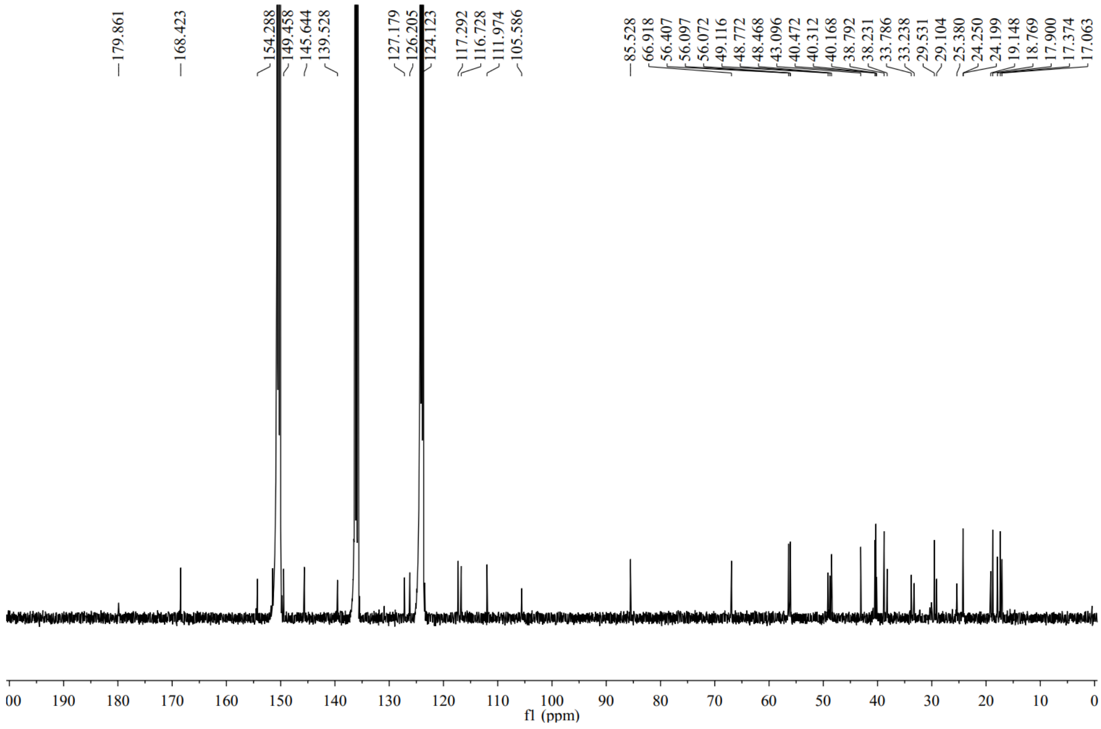

Figure S38. ^{13}C NMR spectrum of **5** (100 MHz, C_5D_5N)

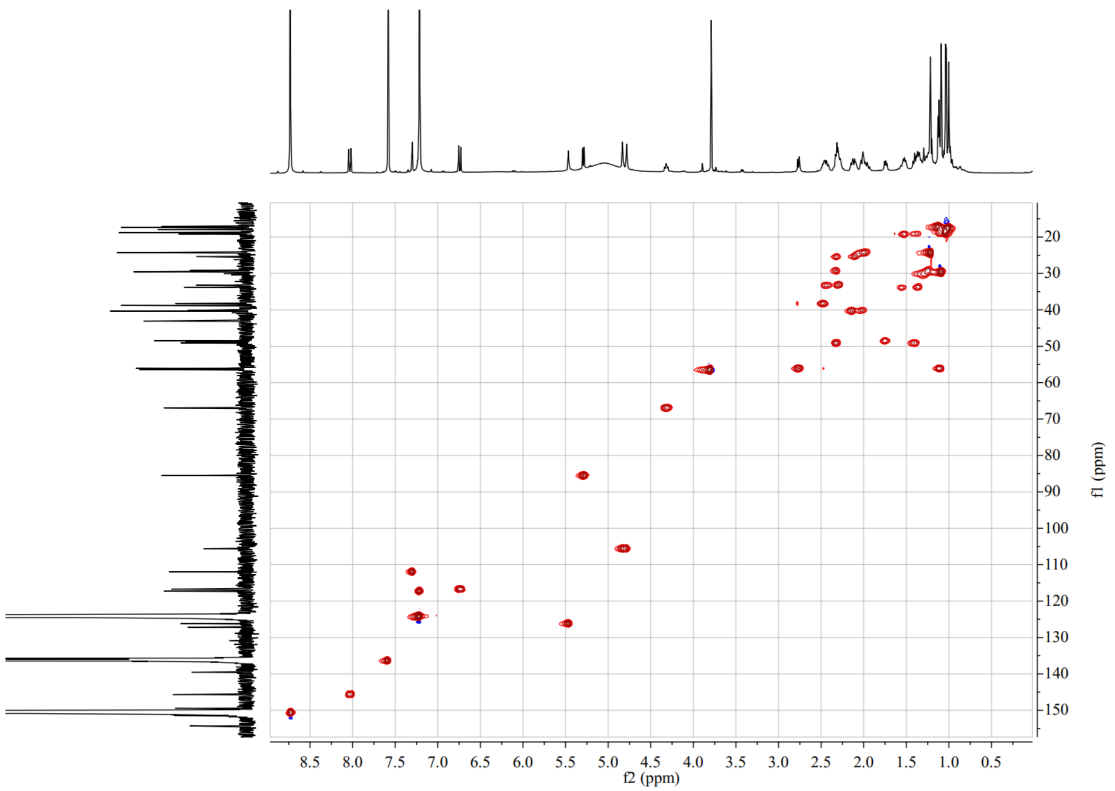

Figure S39. HSQC spectrum of **5** in C_5D_5N

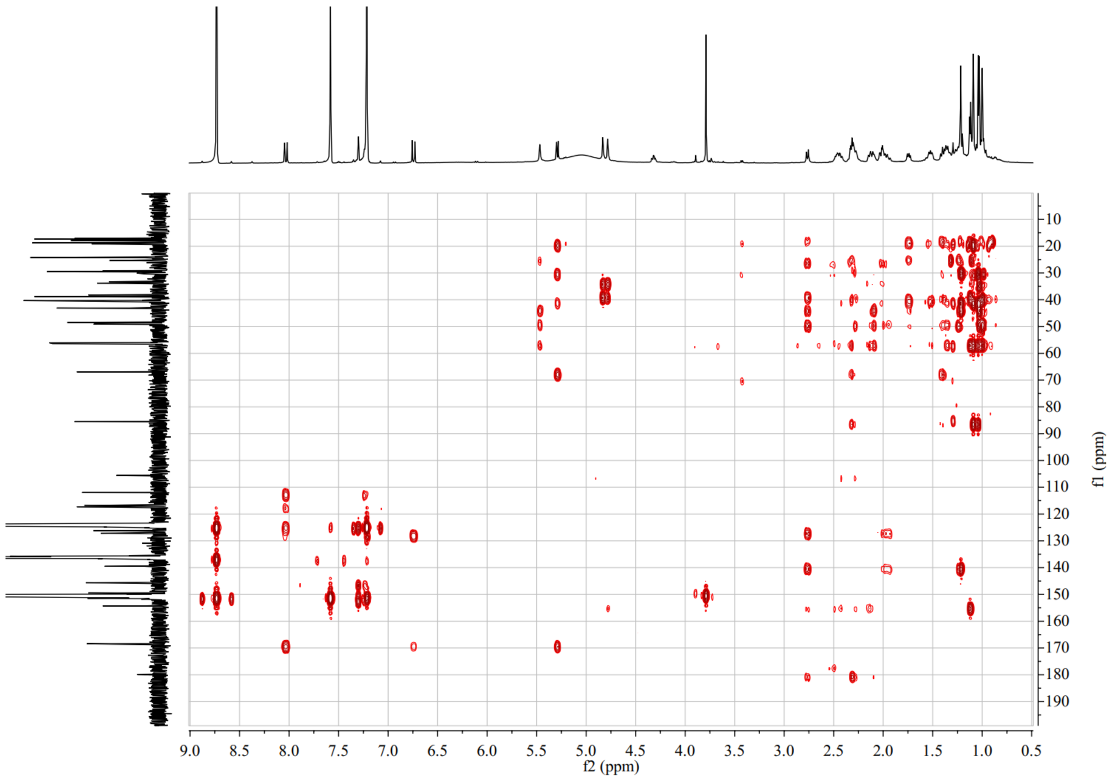

Figure S40. HMBC spectrum of **5** in C_5D_5N

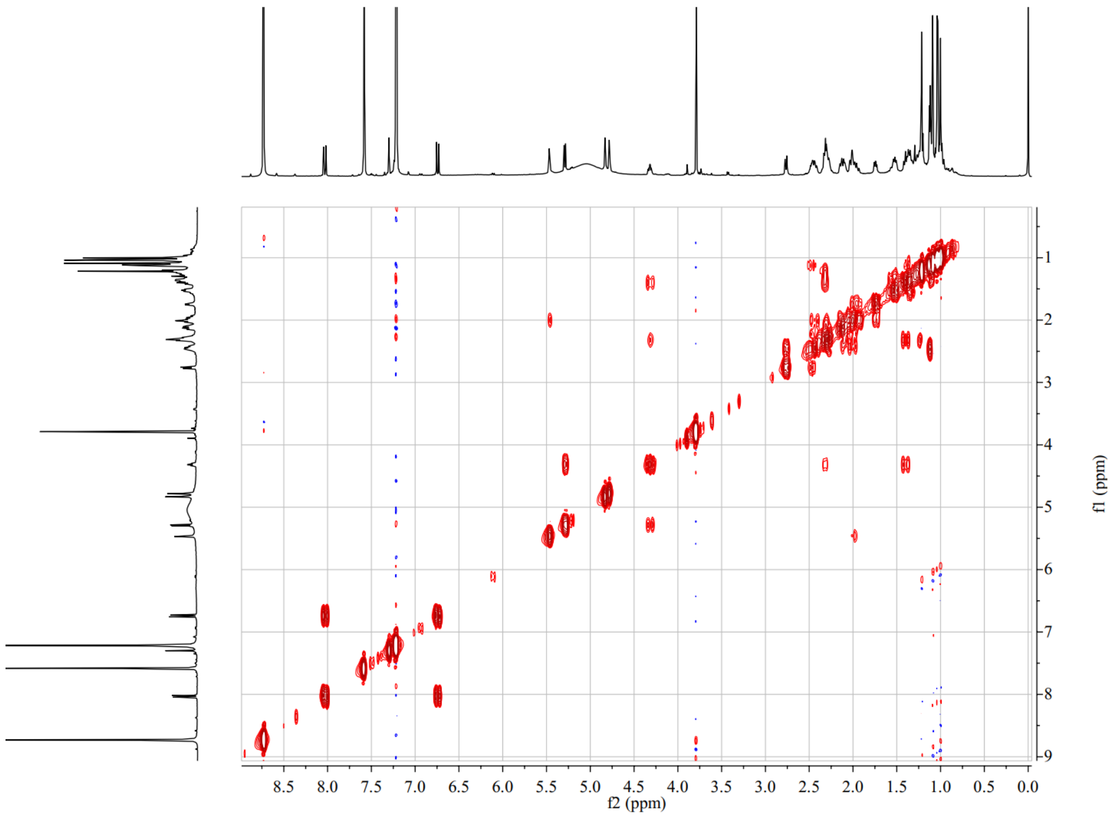

Figure S41. ^{1}H-^{1}H COSY spectrum of **5** in C_5D_5N

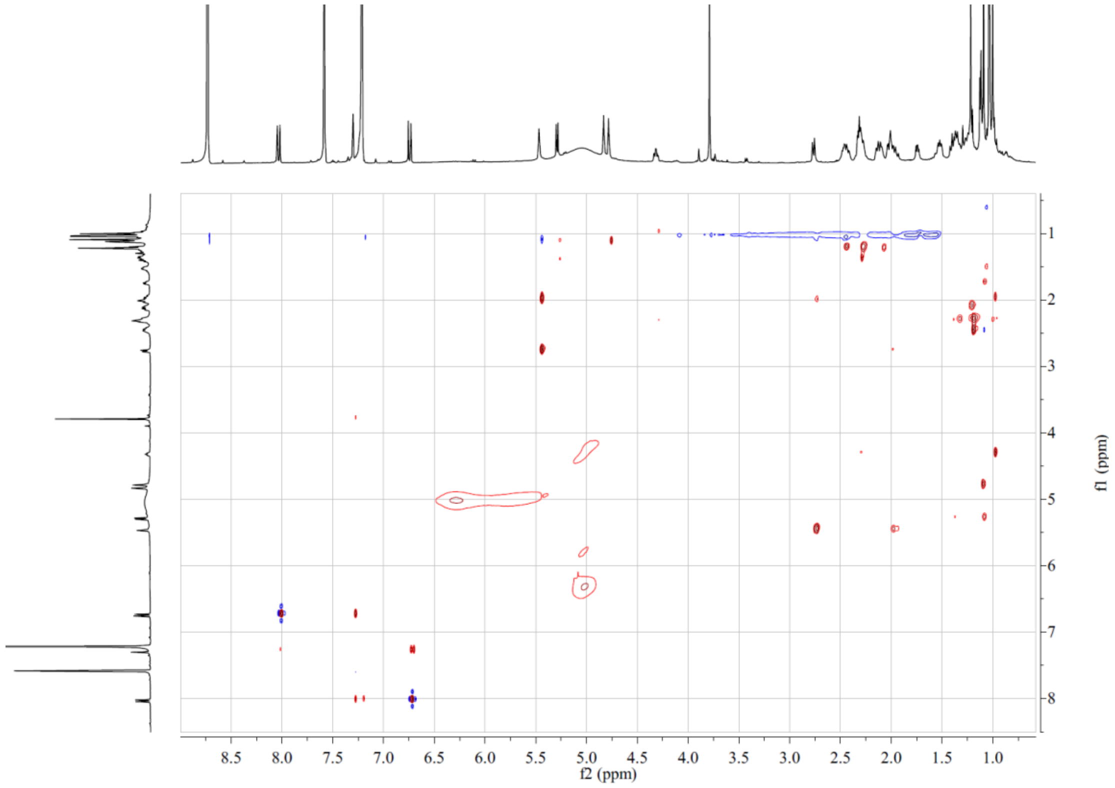

Figure S42. NOESY spectrum of **5** in C_5D_5N

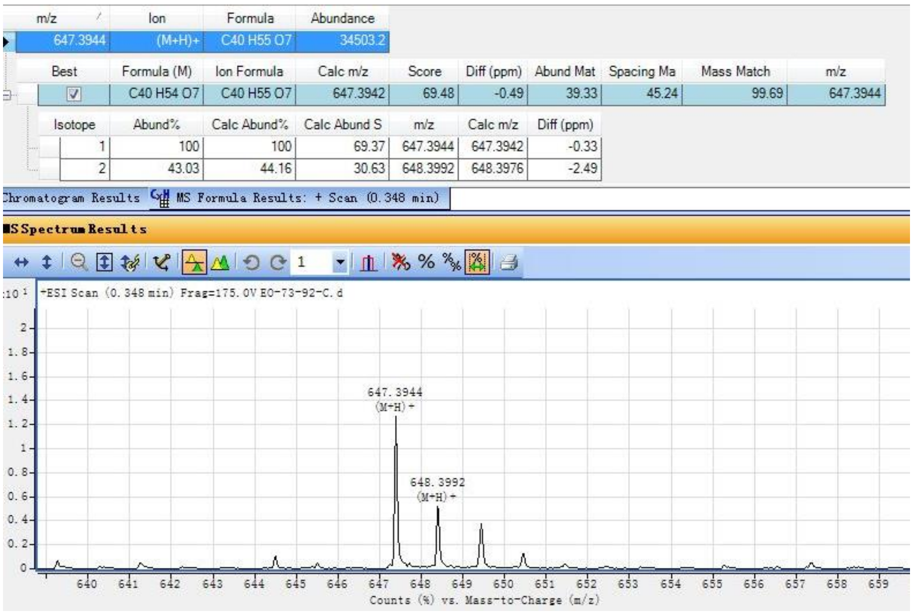

Figure S43. (+)-HRESIMS spectrum of **5**

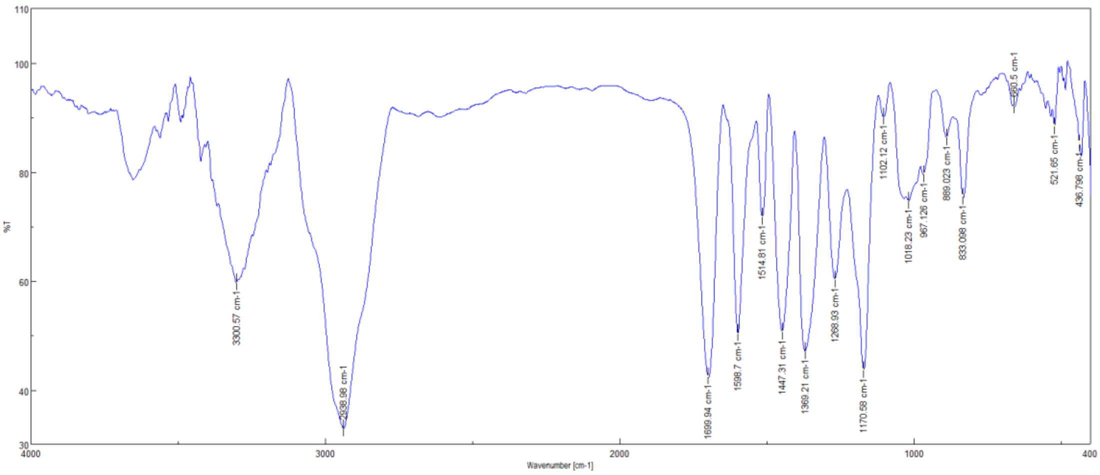

Figure S44. IR spectrum of **5**

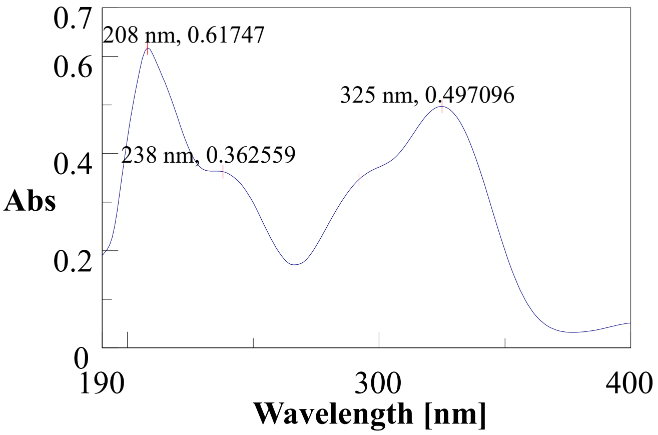

Figure S45. UV spectrum of **5**

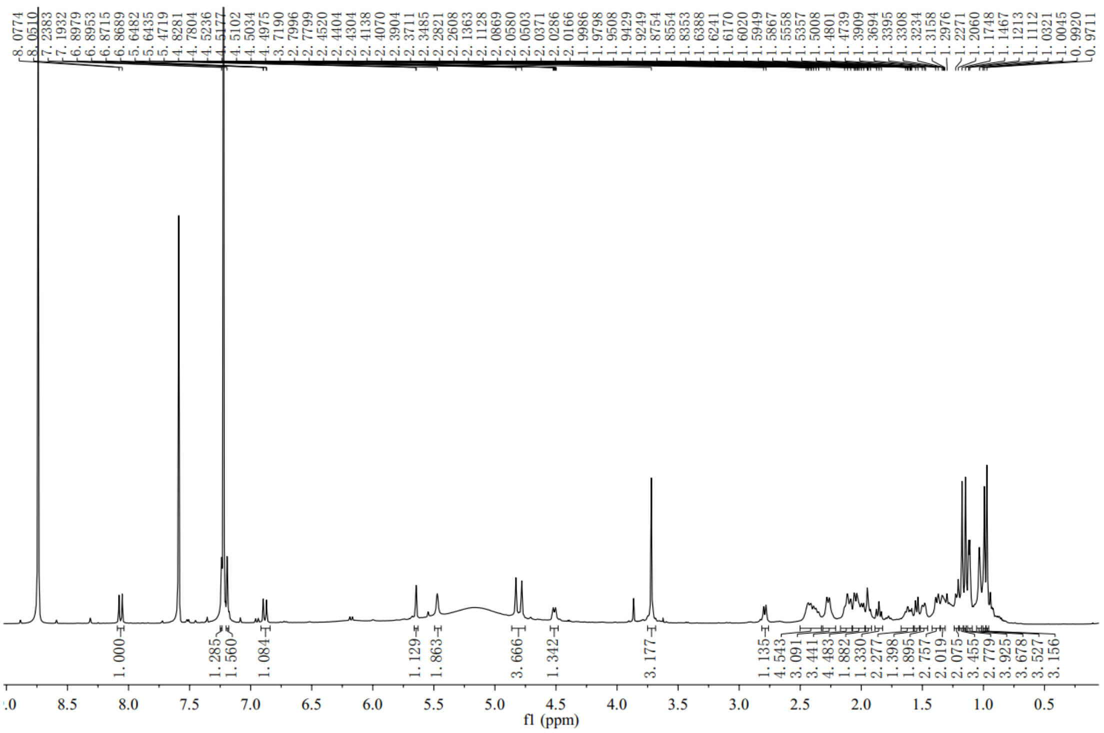

Figure S46. ^{1}H NMR spectrum of **6** (400 MHz, C_5D_5N)

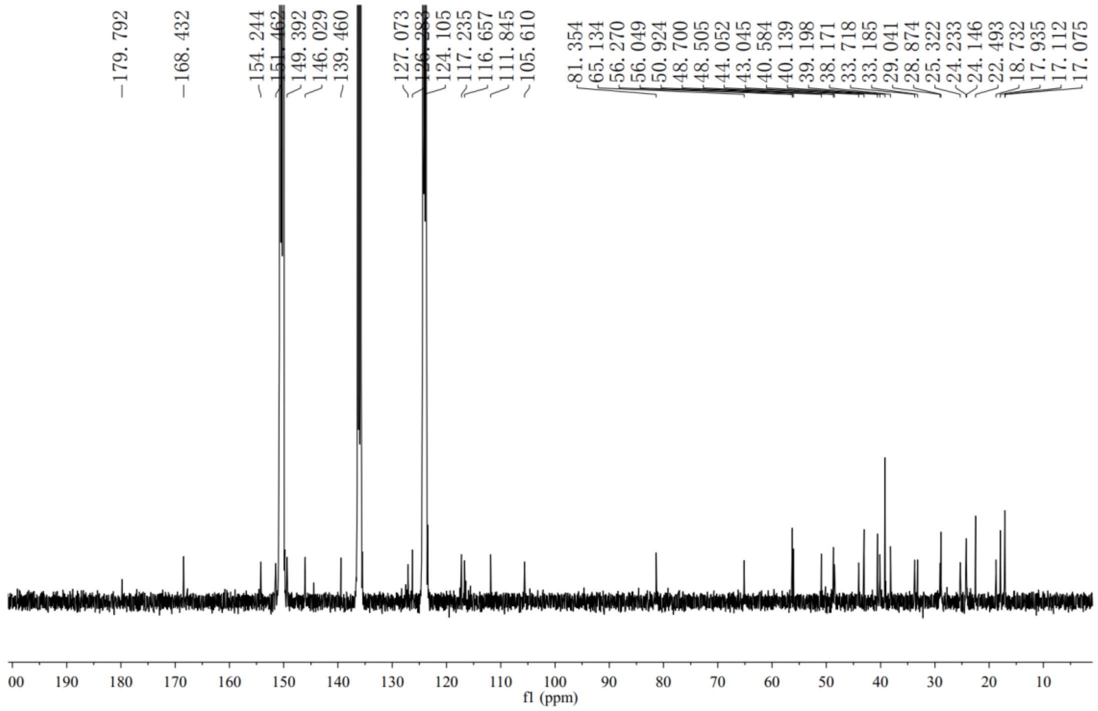

Figure S47. ^{13}C NMR spectrum of **6** (100 MHz, C_5D_5N)

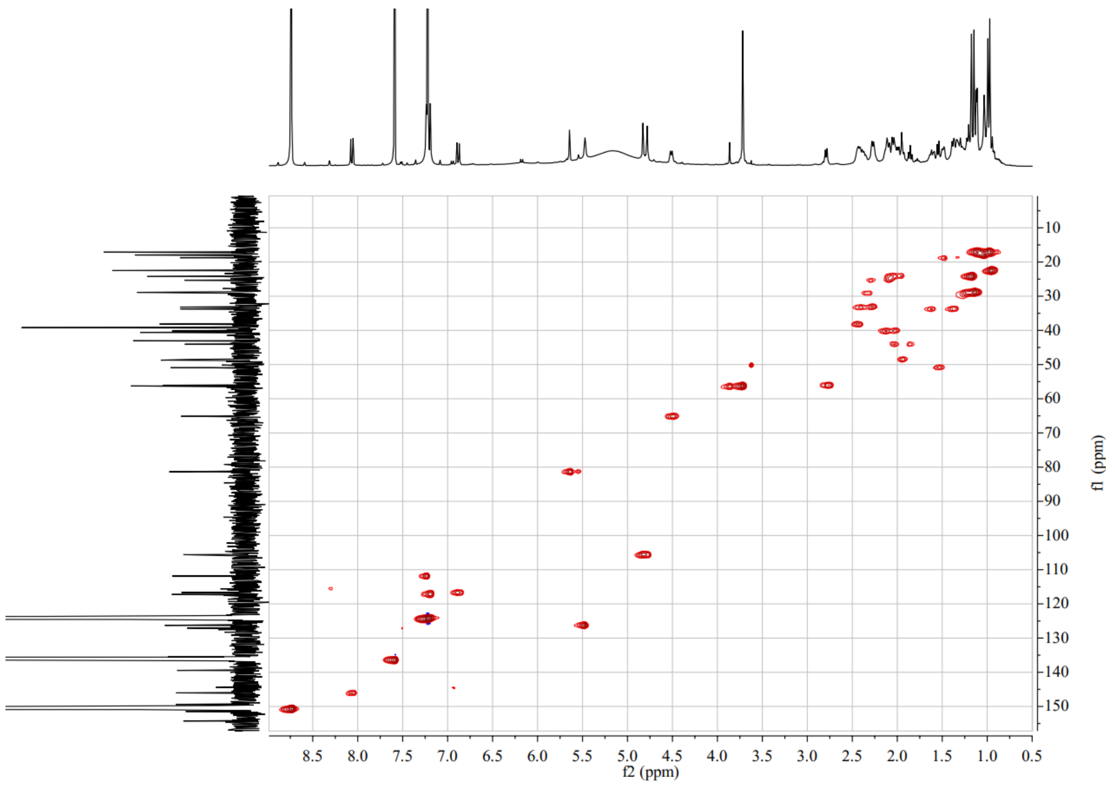

Figure S48. HSQC spectrum of **6** in C_5D_5N

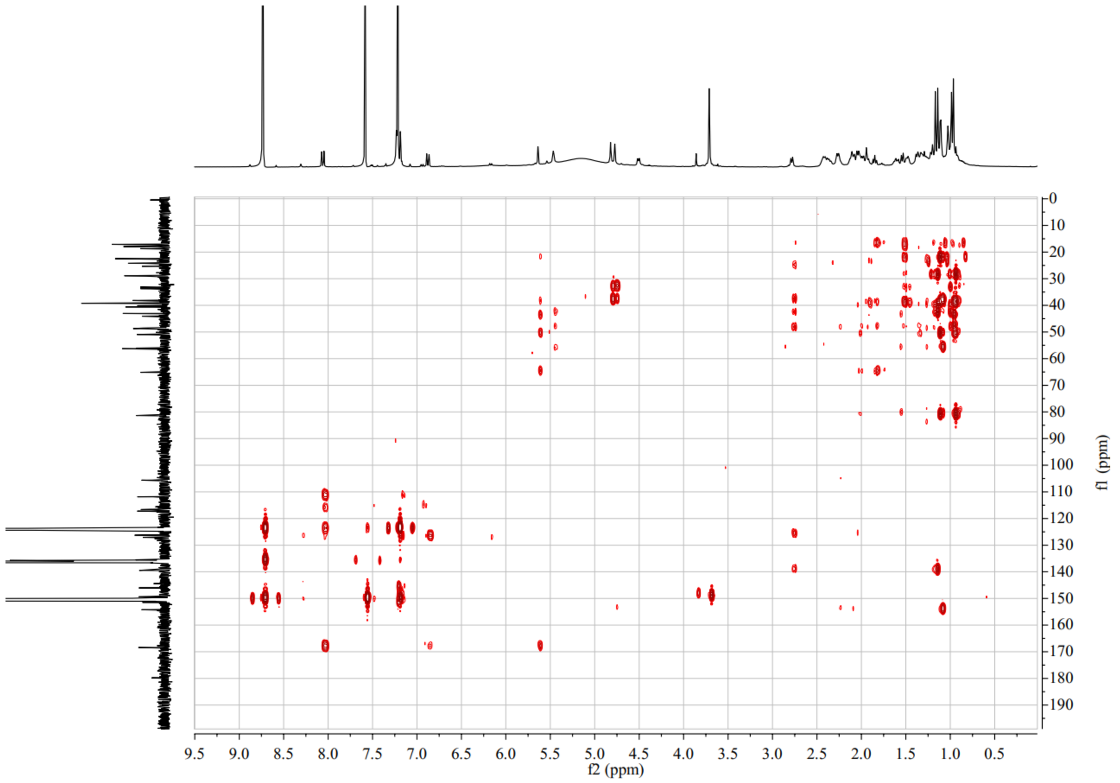

Figure S49. HMBC spectrum of **6** in C_5D_5N

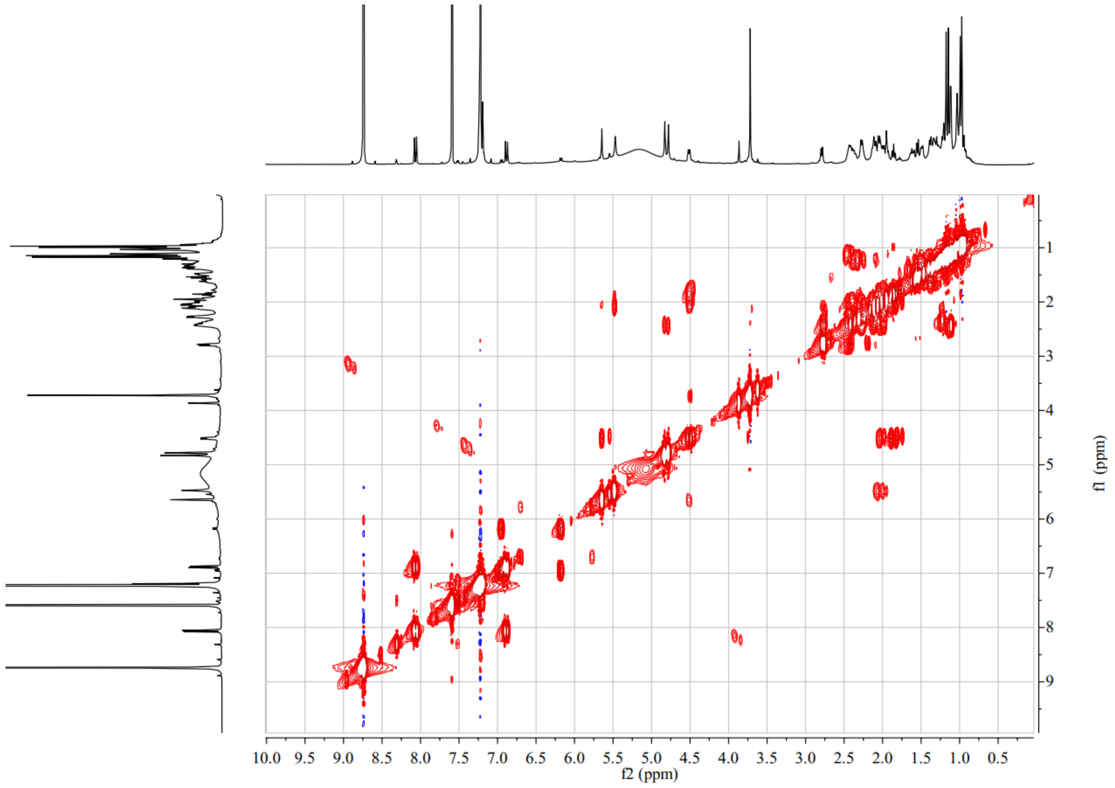

Figure S50. ^{1}H-^{1}H COSY spectrum of **6** in C_5D_5N

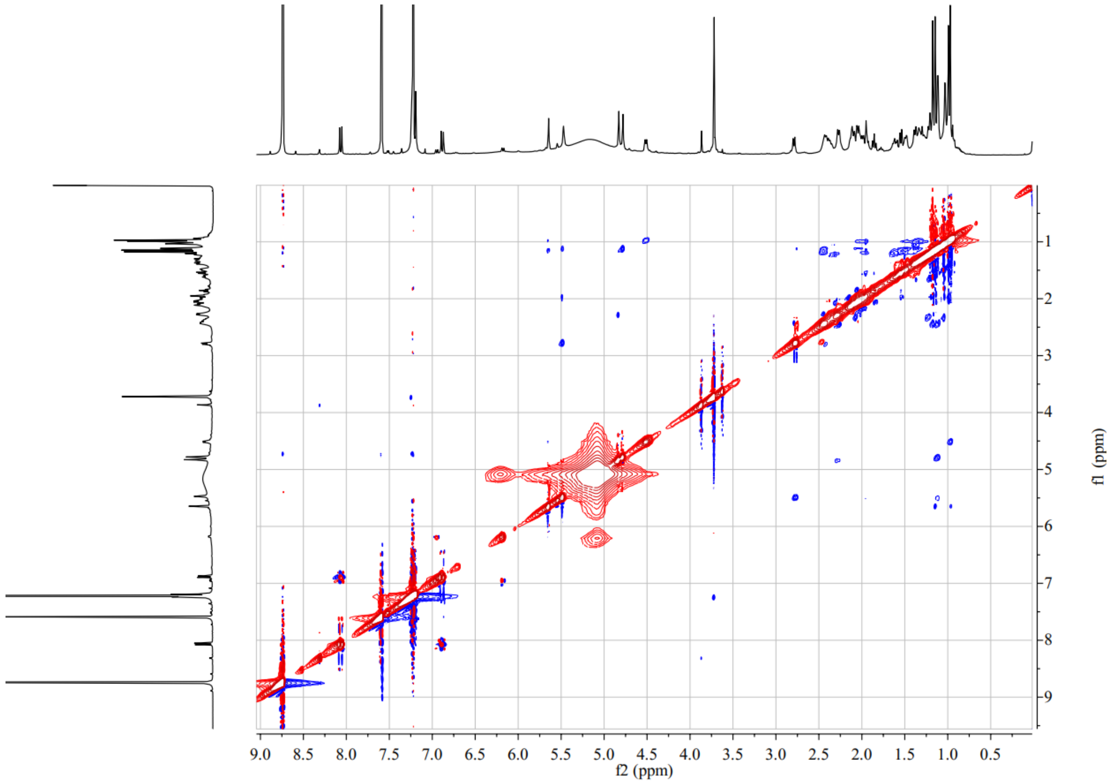

Figure S51. NOESY spectrum of **6** in C_5D_5N

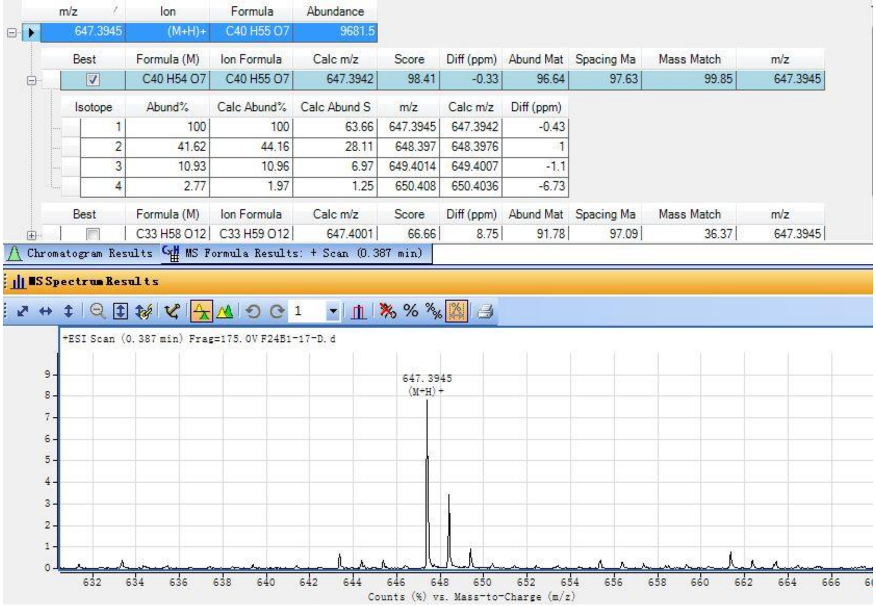

Figure S52. (+)-HRESIMS spectrum of **6**

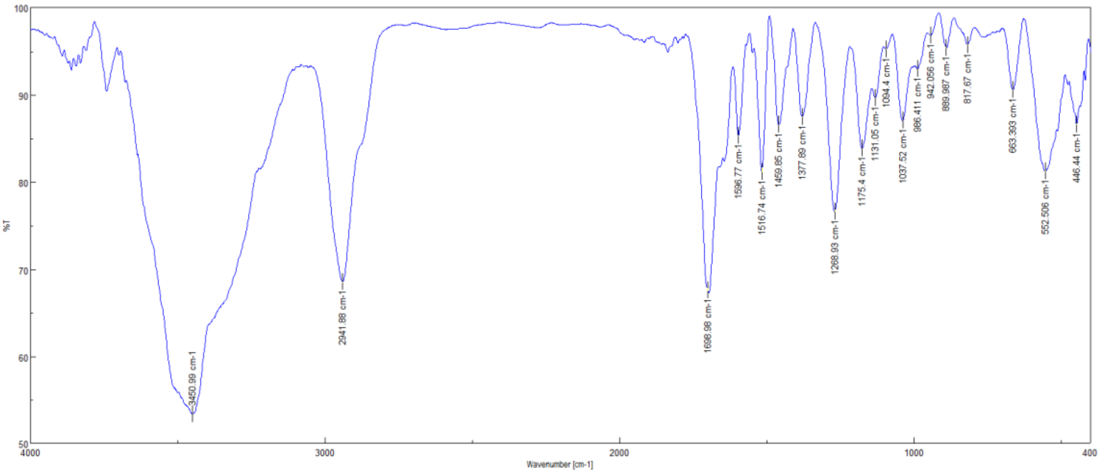

Figure S53. IR spectrum of **6**

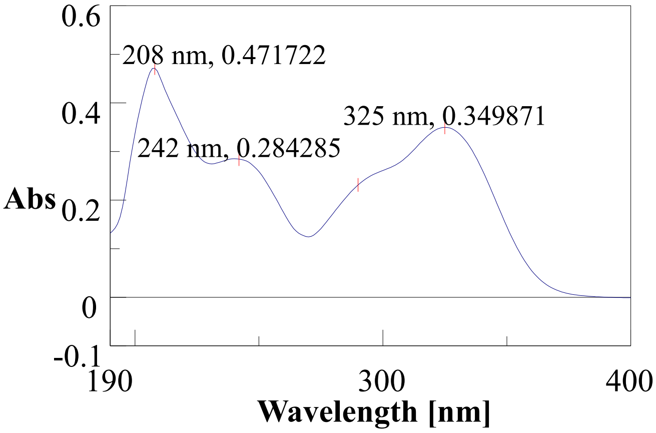

Figure S54. UV spectrum of **6**
